# Discovery of Potent Indolyl-Hydrazones as Kinase Inhibitors for Breast Cancer: Synthesis, X-ray Single-Crystal Analysis, and In Vitro and In Vivo Anti-Cancer Activity Evaluation

**DOI:** 10.3390/ph16121724

**Published:** 2023-12-13

**Authors:** Eid E. Salama, Mohamed F. Youssef, Ahmed Aboelmagd, Ahmed T. A. Boraei, Mohamed S. Nafie, Matti Haukka, Assem Barakat, Ahmed A. M. Sarhan

**Affiliations:** 1 Department of Chemistry, Faculty of Science, Suez Canal University, Ismailia 41522, Egypt; mohamed_gomaa@science.suez.edu.eg (M.F.Y.); ahmed_mohamed@science.suez.edu.eg (A.A.); ahmed_tawfeek83@yahoo.com or ahmed_boraei@science.suez.edu.eg (A.T.A.B.); mohamed_nafie@science.suez.edu.eg (M.S.N.); 2 Department of Chemistry, College of Sciences, University of Sharjah, Sharjah P.O. Box 27272, United Arab Emirates; 3 Department of Chemistry, University of Jyväskylä, P.O. Box 35, FI-40014 Jyväskylä, Finland; matti.o.haukka@jyu.fi; 4 Chemistry Department, College of Science, King Saud University, P.O. Box 2455, Riyadh 11451, Saudi Arabia; 5 Chemistry Department, Faculty of Science, Arish University, Al-Arish 45511, Egypt; ahmed_sarhan252@yahoo.com or asarhan@aru.edu.eg

**Keywords:** indole, hydrazone, MCF7, anticancer, apoptosis, kinase inhibition

## Abstract

According to data provided by the World Health Organization (WHO), a total of 2.3 million women across the globe received a diagnosis of breast cancer in the year 2020, and among these cases, 685,000 resulted in fatalities. As the incidence of breast cancer statistics continues to rise, it is imperative to explore new avenues in the ongoing battle against this disease. Therefore, a number of new indolyl-hydrazones were synthesized by reacting the ethyl 3-formyl-1*H*-indole-2-carboxylate **1** with thiosemicarbazide, semicarbazide.HCl, 4-nitrophenyl hydrazine, 2,4-dinitrophenyl hydrazine, and 4-amino-5-(1*H*-indol-2-yl)-1,2,4-triazole-3-thione to afford the new hit compounds, which were assigned chemical structures as thiosemicarbazone **3**, *bis*(hydrazine derivative) **5**, semicarbzone **6**, Schiff base **8,** and the corresponding hydrazones **10** and **12** by NMR, elemental analysis, and X-ray single-crystal analysis. The MTT assay was employed to investigate the compounds’ cytotoxicity against breast cancer cells (MCF-7). Cytotoxicity results disclosed potent IC_50_ values against MCF-7, especially compounds **5**, **8**, and **12,** with IC_50_ values of 2.73 ± 0.14, 4.38 ± 0.23, and 7.03 ± 0.37 μM, respectively, compared to staurosproine (IC_50_ = 8.32 ± 0.43 μM). Consequently, the activities of compounds **5**, **8**, and **12** in relation to cell migration were investigated using the wound-healing test. The findings revealed notable wound-healing efficacy, with respective percentages of wound closure measured at 48.8%, 60.7%, and 51.8%. The impact of the hit compounds on cell proliferation was assessed by examining their apoptosis-inducing properties. Intriguingly, compound **5** exhibited a significant enhancement in cell death within MCF-7 cells, registering a notable increase of 39.26% in comparison to the untreated control group, which demonstrated only 1.27% cell death. Furthermore, the mechanism of action of compound **5** was scrutinized through testing against kinase receptors. The results revealed significant kinase inhibition, particularly against PI3K-α, PI3K-β, PI3K-δ, CDK2, AKT-1, and EGFR, showcasing promising activity, compared to standard drugs targeting these receptors. In the conclusive phase, through in vivo assay, compound **5** demonstrated a substantial reduction in tumor volume, decreasing from 106 mm³ in the untreated control to 56.4 mm³. Moreover, it significantly attenuated tumor proliferation by 46.9%. In view of these findings, the identified leads exhibit promises for potential development into future medications for the treatment of breast cancer, as they effectively hinder both cell migration and proliferation.

## 1. Introduction

Globally, cancer stands as the foremost contributor to mortality, with breast cancer ranking among the leading causes of death in women. The complexity of this ailment poses a significant challenge to medical therapy. Despite their capacity to eliminate cancer cells, conventional anticancer treatments often induce a multitude of adverse effects, proving detrimental to healthy tissues. Traditional antineoplastic drugs, aimed at inhibiting specific molecules fostering tumor growth, commonly result in side effects. In response, scientists are actively exploring novel anticancer drugs, engaging in the design and discovery of new compounds tailored for treating various cancer types. This pursuit aims to identify targeted therapies that can potentially offer more effective and less harmful treatment options [1,2]. Kinases, constituting the sixth largest class of proteins in the human body [3,4,5], play a crucial role in cellular function. Inhibitors of kinases are indispensable for maintaining proper cellular activities, as they modulate kinase dysregulation associated with various diseases and disorders, including cancer, inflammatory conditions, and responses to external stimuli. Through the regulation of protein kinases, these inhibitors effectively impede the growth of their substrates, thereby exerting control over the viability and proliferation of cells [6,7,8,9]. 

Various therapeutic targets for kinase inhibitors exist, encompassing EGFR, CDK, AKT, PI3K, and other specific targets [10,11,12]. Indole-containing compounds have garnered prolonged attention from researchers and evolved into a dynamic field of study. The indole moiety demonstrates a high affinity for binding to several receptors, paving the way for the development of new bioactive medications. Its widespread utilization in target-based discovery and the design of anticancer drugs is well-documented [13,14,15,16,17,18].

**Figure 1:**
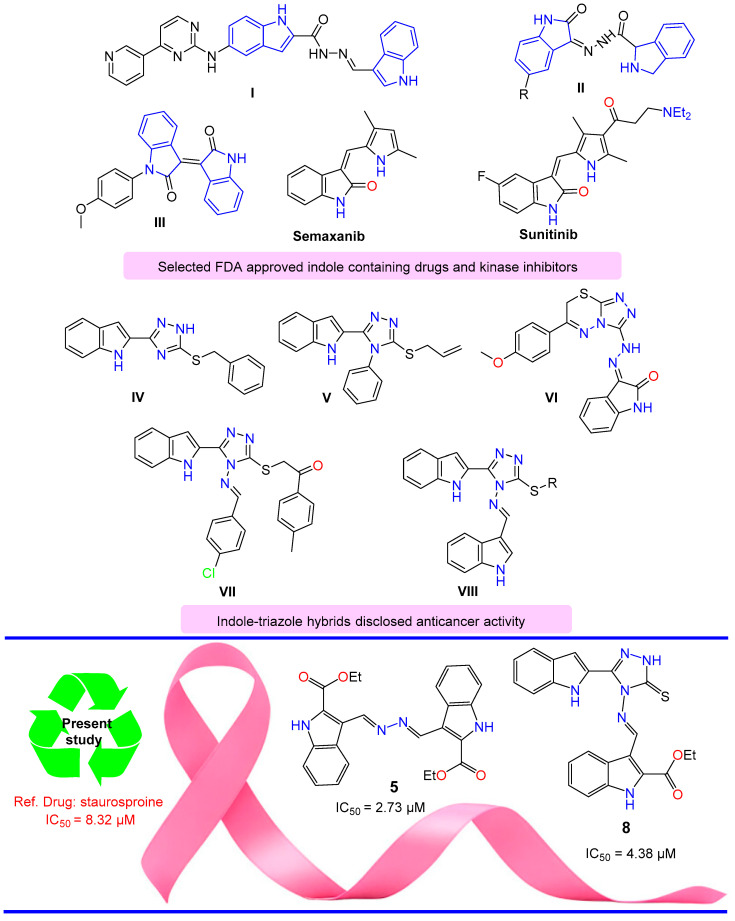
Selected indole structures and drugs that possess anticancer activity.

*N*’-Methylene-5-((4-(pyridin-3-yl)pyrimidin-2-yl)amino)1*H*-indole-2-carbohydrazide moiety showed CDK9 inhibitory activity for cancer therapy treatment, according to Hu et al. [19]. Some representative examples discussed, such as compound **I**, were assessed, and it was revealed that they showed potential against CDK9 inhibition. Al-warhi et al. reported that oxindole **II** displayed anti-tumor activity targeting the CDK4 inhibitor [20]. *N*-substituted iso-indigo compounds were designed, synthesized, and biologically evaluated by Zhao et al. as inhibitors of cyclin-dependent kinase 2 (CDK2). Iso-indigo compound **III** was found to stop the S-phase of the cell cycle [21] (Figure 1). 

Sunitinib is used for the treatment of gastrointestinal stromal tumors (GIST) and advanced renal cell carcinoma (RCC) [22,23,24,25]. Semaxanib is a tyrosine kinase inhibitor drug that is used in cancer therapeutics [26]. Indole-triazole alkylated system (Figure 1) displayed significant anti-cancer activity. For example: 3-benzylsulfanyl-5-(1*H*-indol-2-yl)-2*H*-1,2,4-triazole **IV** showed promising antiproliferative activity against HEPG-2 and MCF-7 cancer cell lines [27]; 3-(allylsulfanyl)-4-phenyl-5-(1*H*-indol-2-yl)-1,2,4-triazole **V** and its analogues showed interesting anti-proliferative potential against breast cancer [28]; substituted 3-(triazolo-thiadiazin-3-yl)-indolin-2-one derivatives **VI** displayed dual inhibition activity for c-Met (a receptor tyrosine kinase) and VEGFR2 enzymes, with an effective anti-proliferative potency against different subpanels of the most NCI 58 tumor cell lines [29]; and indole-triazole hybrid **VII** and its analogues revealed a potent inhibition against vascular endothelial growth factor receptor-2 (VEGFR-2), with potential anti-renal cancer activity [30]. Alkylated indolyl-triazole Schiff bases **VIII** targeted breast cancer via VEGFR2 tyrosine kinase inhibition [31]. 

Building upon the findings from the aforementioned studies, the conceptualization of synthesizing novel compounds that incorporate ester and azomethine groups, along with an indole scaffold in a single compound, is expected to yield potent anticancer medicines. This anticipation stems from the potential of these compounds to function as both hydrogen bond donors and/or acceptors upon interaction with receptors.

## 2. Results and Discussion

### 2.1. Synthesis

**Scheme 1:**
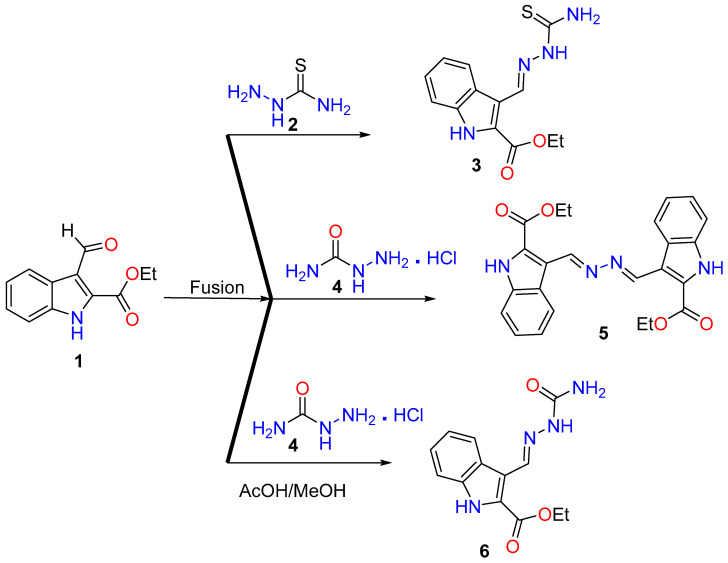
Reaction of thiosemicarbazide **2** and semicarbazide.HCl **4** with ethyl 3-formyl-1*H*-indole-2-carboxylate **1**.

Condensation of thiosemicarbazide **2** with ethyl 3-formyl-1*H*-indole-2-carboxylate **1** by fusion for 5 min led to the formation of the thiosemicarbazone derivative **3**. Under the same fusion condition, the condensation of **1** with semicarbazide.HCl **4** interestingly afforded *bis*-ester derivative **5**. Through the reaction of **1** with semicarbazide.HCl **4** under reflux in AcOH/MeOH, semicarbazone derivative **6** was obtained (Scheme 1). 

**Scheme 2:**
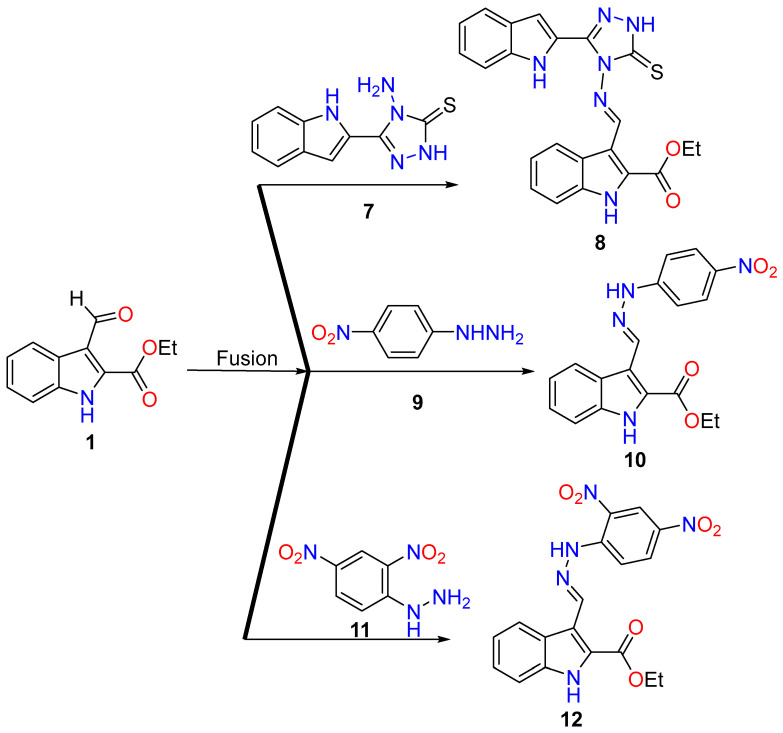
Synthesis of hydrazones **8**, **10**, and **12**.

Under fusion conditions, the reaction between ethyl 3-formyl-1*H*-indole-2-carboxylate **1** with amine functionality derivatives, such as 4-amino-5-(1*H*-indol-2-yl)-1,2,4-triazole-3-thione **7**, 4-nitrophenyl hydrazine **9,** and 2,4-dinitrophenyl hydrazine **11**, resulted in the formation of condensed hydrazones **8**, **10**, and **12**, respectively (Scheme 2). The structural assignment for the newly synthesized hits was established through a comprehensive set of spectroscopic tools (see Section 3), which included nuclear magnetic resonance (NMR), mass spectrometry (MS), CHN analysis, and single-crystal X-ray diffraction analysis.

### 2.2. X-ray Single-Crystal Analysis for Compounds ***3*** and ***5***

**Figure 2:**
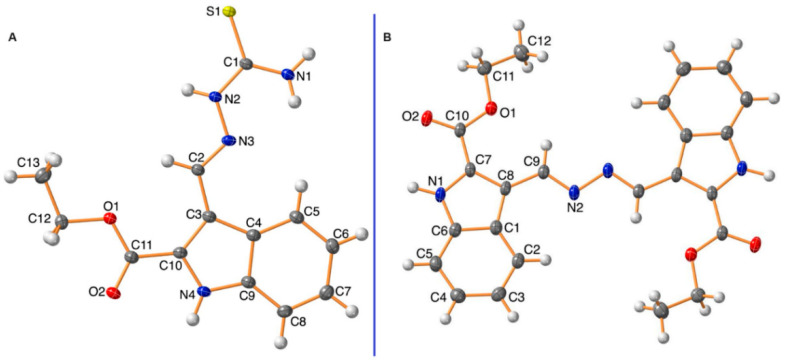
Ortep representation of **3** (**A**) and **5** (**B**).

Using single-crystal X-ray analysis, structures of compounds **3** and **5** were conclusively confirmed. The unit cell parameters of compound **3** (*a* = 9.23490(10) Å, *b* = 19.4168(2) Å, *c* = 7.89280(10) Å, and *V* = 1374.36(3) Å^3^) that crystallized in monoclinic space group *P2_1_/c* and compound **5** (*a* = 5.4774(3) Å, *b* = 9.2031(5) Å, *c* = 10.6234(8) (10) Å, and *V* = 531.26(6) show the Å^3^ that crystallized in the triclinic space group *P* *1* (Table 1). The crystal structure (Figure 2) revealed that compound **3** was a thiosemicarbazone structure, while compound **5** was a *bis*-hydrazino derivative. 

### 2.3. Biology

#### 2.3.1. MTT Assay for the Synthesized Compounds

The produced compounds were examined for their cytotoxicity against MCF-7 breast cancer cells using the MTT assay. As summarized in Table 2, they demonstrated potent IC_50_ values against MCF-7, especially compounds **5**, **8**, and **12**, with IC_50_ values of 2.73 ± 0.14, 4.38 ± 0.23, and 7.03 ± 0.37 μM, compared to staurosproine, with an IC_50_ value of 8.32 ± 0.43 μM, while compounds **1**, **3**, and **6** exhibited promising cytotoxicity, with IC_50_ values of 19.7 ± 2.31, 10.2 ± 0.53, and 9.42 ± 0.57 μM, respectively. Compound **10** exhibited moderate cytotoxicity, with a high concentration of IC_50_ (25.4 ± 1.54 μM). 

#### 2.3.2. Wound-Healing Activity

**Figure 3:**
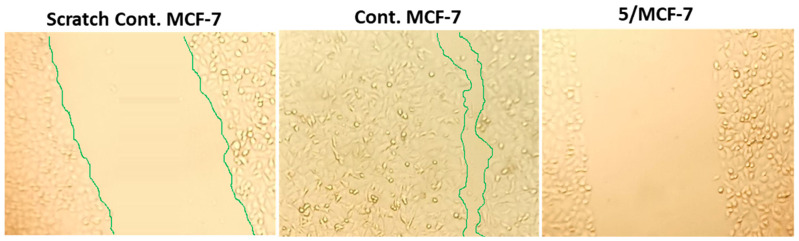
Migration of MCF-7 cells treated with compound **5** for 72 h observed under a light microscope as detected by the wound-healing assay.

As shown in Table 3 and Figure 3, the wounded area between cell layers following a scratch was partially filled by migrating MCF-7 control cells (94.07% wound closure), while treatments of compounds **5**, **8**, and **12** significantly inhibited wound-healing activity, with percentages of wound closure of 48.88, 60.74, and 51.85%, respectively, compared to control. 

#### 2.3.3. Apoptotic Induction Activity

To investigate the apoptotic activity of compounds **5**, **8**, and **12**, flow cytometric evaluation of Annexin V/PI staining was utilized to examine apoptotic cell death in untreated and treated MCF-7 cells. Table 4 shows that compound **5** dramatically increased cell death in MCF-7 cells by 39.26% (29.35% for apoptosis and 9.91% for necrosis), compared to the untreated control group, which increased it by 1.27% (0.4% for apoptosis and 0.87% for necrosis). Additionally, compounds **8** and **12** caused total cell death by 24.4% and 37%, with apoptosis ratios of 15.72% and 21.0%, respectively. 

**Figure 4:**
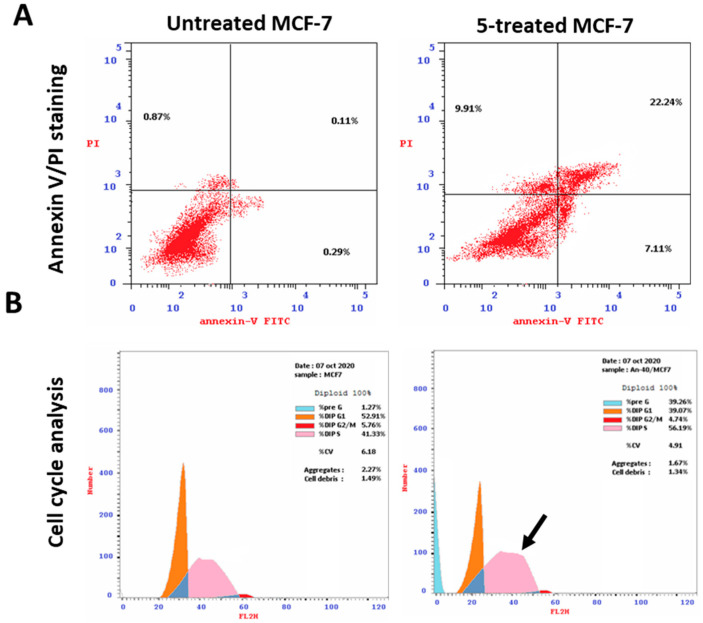
Analysis using flow cytometry. Upper panel (**A**): Annexin V/PI staining for evaluating necrosis and apoptosis, Q1;eEarly apoptosis is Q4, and late apoptosis is Q2. The DNA content histograms of untreated and **5**-treated MCF-7 cells at each phase, “Pre-G, G1, G2/M, S” phases, with an IC_50_ value of 2.73 µM, 48 h, are displayed in the lower panel (**B**).

After being treated with a cytotoxic chemical, the cell population in each cell phase was then ascertained by DNA flow cytometry. Compound **5** increased the S-phase cell population by 56.2%, compared to control, which increased it by 41.33%, as Figure 4 illustrates, whereas cells in other phases decreased negligibly. Consequently, compound **5** stopped MCF-7 cells from proliferating at the S-phase by inducing apoptosis.

#### 2.3.4. Kinase-Inhibition Activity

To highlight their effective molecular target, the most cytotoxic and apoptotic compound **5** was screened for its activity towards a panel of kinase activities, including PI3K-*α*, PI3K-*β*, PI3K-*δ*, CDK2, AKT-1, and EGFR, compared to their standard drugs. It caused promising kinase inhibitory activities, as summarized in Table 5. Interestingly, compound **5** exhibited significant inhibitory potential against PI3K-*α* and showed selectivity, with a 4.92-fold higher potency than LY294002. However, in the cases of PI3K-*β* and PI3K-*δ*, compound **5** demonstrated lower activity compared to LY294002. Moreover, compound **5** (IC_50_ = 0.156 ± 0.01 μM) showed a reactivity profile against CDK2 closer to the standard drug erlotinib (IC_50_ of 0.173 ± 0.01 μM). On the other hand, compound **5** (IC_50_ = 0.602 ± 0.03 μM) demonstrated lower reactivity towards AKT-1, compared to the standard drug A-674563 (IC_50_ of 0.26 ± 0.01 μM). Finally, compound **5** (IC_50_ = 0.058 ± 0.029 μM) demonstrated lower reactivity against EGFR, compared to the standard drug erlotinib (IC_50_ of 0.038 ± 0.019 μM). Compound **5** was found to possess the potential for inhibiting multiple kinases. 

#### 2.3.5. In Vivo (SEC-Bearing Mice)

**Figure 5:**
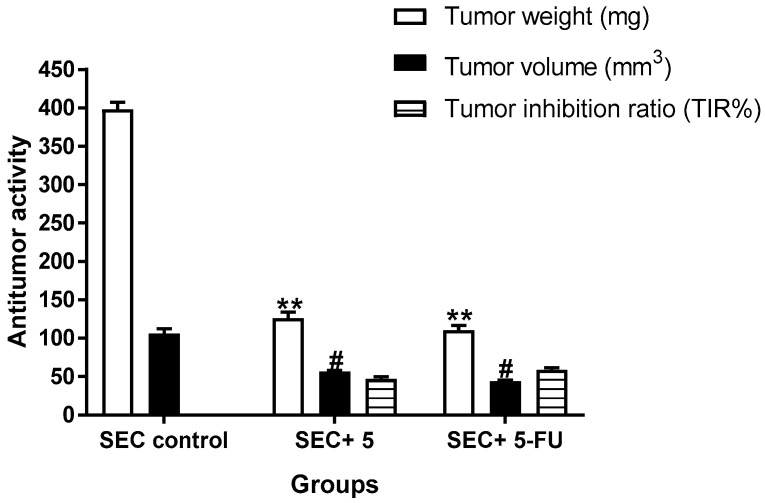
Measurements of antitumor potentiality in the SEC-bearing mice treated with compound **5** and 5-FU. “Mean ± SD values of mice in each group (*n* = **6**)”. “** Values are highly significantly different (*p* ≤ 0.01) between treated and SEC control”, while “^#^ values are significantly different (*p* ≤ 0.05) between treated SEC and SEC control mice using the un-paired test in GraphPad prism”. TIR% = C − T/C × 100.

A solid Ehrlich carcinoma cell was implanted, and **5** was injected intraperitoneally (IP) throughout the experiment to confirm its anticancer efficacy, as shown in Figure 5, which summarizes the main findings of the antitumor activity experiments. As a result, tumor proliferation revealed an increase in solid tumor mass of approximately 398.1 mg, which is related to tumor proliferation. Following treatment with **5**, the solid tumor mass decreased to 126.5 mg, compared to 110 mg in the 5-FU treatment. As a result, treatments with **5** considerably reduced tumor volume from 10^6^ mm^3^ in the untreated control to 56.4 mm^3^ and significantly decreased tumor proliferation by 46.9%, while 5-FU reduced tumor volume to 43.7 mm^3^ and inhibited tumor development by 58.8%.

#### 2.3.6. Molecular Docking

**Figure 6:**
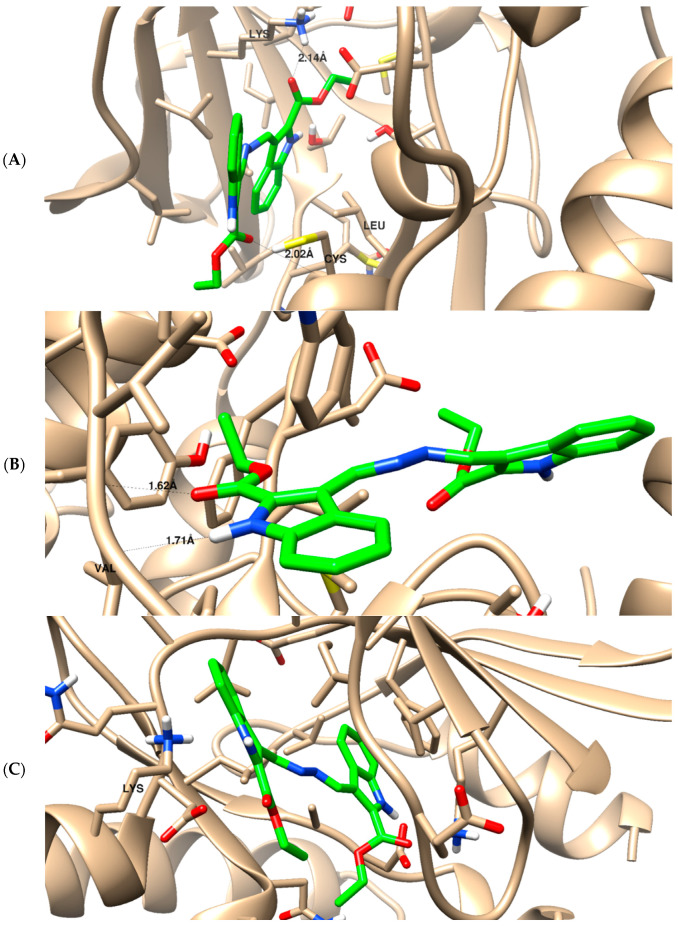
The binding disposition of docked compound **5** with ligand–receptor interactions inside the EGFR receptor (**A**), phosphoinositide 3-kinase (**B**), and cyclin-dependent kinase (**C**). Visualization was carried out using Chimara-UCSF.

To illustrate the virtual mechanism of binding towards the EGFR, PI3K, and CDK2 binding sites, molecular docking research was carried out. As seen in Figure 6, compound **5** was properly docked inside the protein active sites of EGFR (A), PI3K (B), and CDK2 (C), with binding energies of −23.15, −21.32, and −23.44 Kcal/mol, and it formed good binding interactions with their active sites. Compound **5** exhibited strong binding interactions with the amino acids Lys721, Cys773, and Leu694 inside EGFR. It formed two H-bond interactions with Val882 inside the PI3K active site, and it formed two arene–cation interactions with Lys89 inside the CDK2 active site like the co-crystallized ligands. These outcomes corroborated the kinase inhibition experiment findings. Previous literature reported the downstream inhibition pathway of EGFR/PI3K/AKT, which is linked to CDK2 inhibition, as a promising target for inducing apoptosis in cancer cells [32]. 

**Figure 7:**
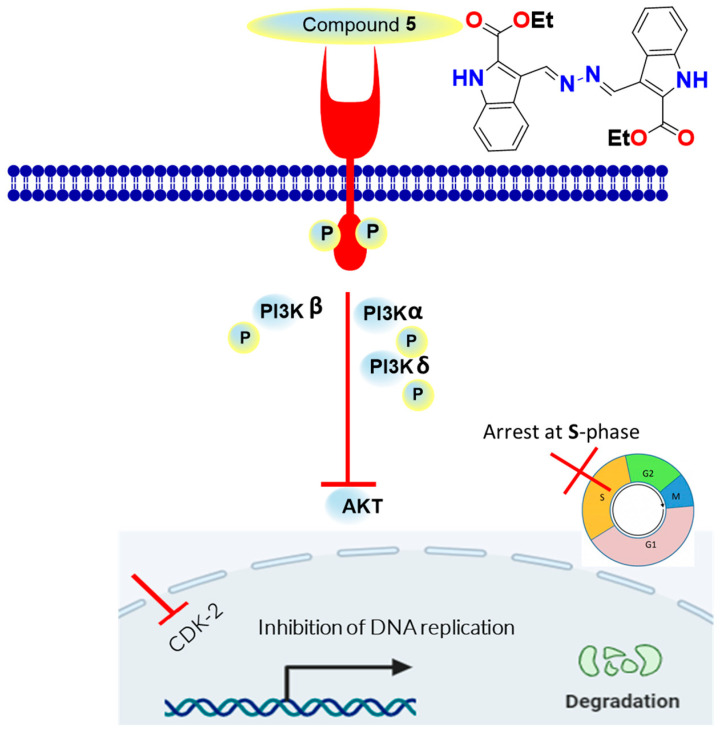
Schematic diagram for the mechanistic pathway for EGFR/PI3K/CDK2 inhibition as apoptosis inducers.

As summarized in Figure 7, compound **5**, as an indolyl-hydrazone derivative, induced potent cytotoxicity against MCF-7 as an apoptosis inducer through the downstreaming pathway of EGFR/PI3K/AKT and CDK2 inhibition. The effective pathway induced cell cycle arrest at the S-phase, and it led to apoptosis in the MCF-7 cells. EGFR, and its downstreaming pathway is considered one of the promising effective pathways for cancer treatment, and our results agreed with previous reported studies for the same compounds’ scaffold affecting cytotoxic activities through apoptosis [33,34,35]. 

#### 2.3.7. SAR

**Figure 8:**
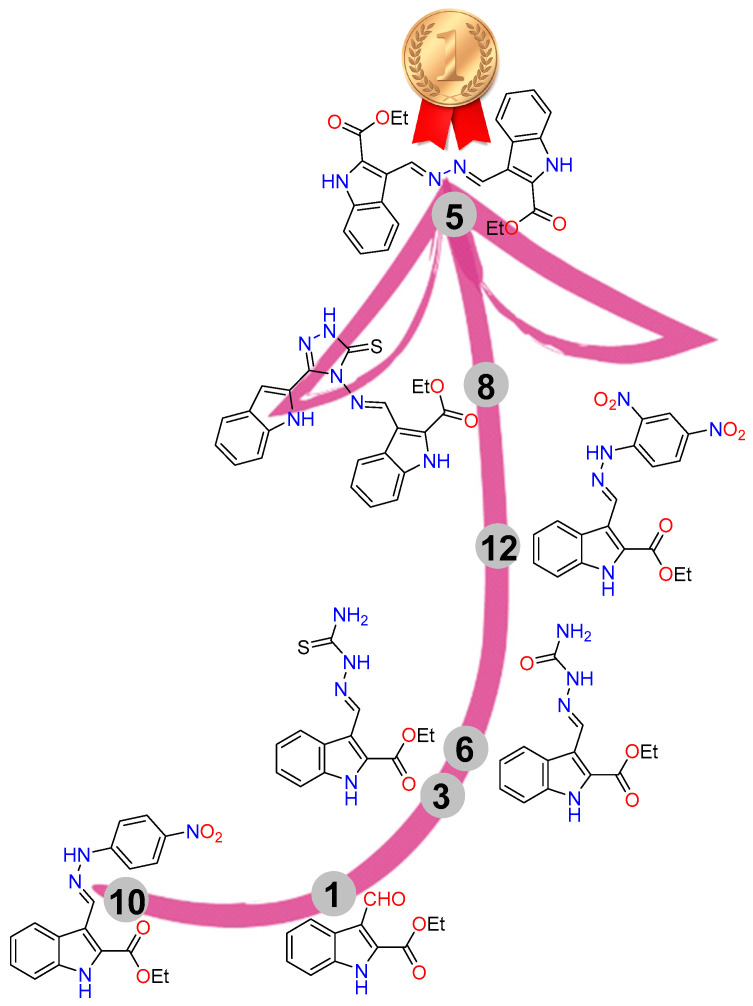
SAR for the synthesized compounds.

The structure–reactivity relationship of the synthesized compounds is summarized as follows in Figure 8: The hydrazone derivative **10**, featuring a *p*-nitro group-substituted benzene ring, exhibited the lowest reactivity, with an IC_50_ value of 25.4 ± 1.54 μM. In contrast, the aldehyde-based indole derivative **1**, the starting material, demonstrated better reactivity, with an IC_50_ value of 19.7 ± 2.31 μM. The thiosemicarbazide **3** and its isosteric semicarbazide **6** enhanced reactivity, with IC_50_ values of 9.42 ± 0.57 and 10.2 ± 0.53 μM, respectively. The presence of two nitro groups on the substituted benzene ring of hydrazone **12** significantly improved reactivity, with an IC_50_ value of 7.03 ± 0.37 μM, due to the high electron-withdrawing group effect. The introduction of the thio-triazole indole-based Schiff base **8** significantly increased activity (IC_50_ = 4.38 ± 0.23 μM) up to 1.9-fold higher than the reference drug, while the symmetrical *bis*-esters azine **5** emerged as the most potent compound in inhibiting breast cancer cells, with an IC_50_ of 2.73 ± 0.14 μM, 3-fold more potent than the standard drug staurosporine (IC_50_ = 8.32 ± 0.43 μM).

## 3. Materials and Methods

### 3.1. Chemistry

#### 3.1.1. General 

The values for the melting points were uncorrected and were determined in open capillaries using a Temp-melt II melting point equipment. On silica gel 60 (230–400 mesh ASTM), flash chromatography was carried out. On silica gel 60 F254 aluminum plates (E. Merck, layer thickness 0.2 mm), thin-layer chromatography (TLC) was performed. The spots were found using a UV lamp. Using DMSO-d_6_ and CDCl_3_ as solvents, the ^1^H and ^13^C-NMR spectra were captured on Bruker instruments at 400 MHz for ^1^H NMR and 101 MHz for ^13^C NMR, respectively. Using KBr and a PerkinElmer 1430 ratio-recording infrared spectrophotometer, Bruker’s Fourier-transform infrared (FT-IR) spectrophotometry was used to record the IR spectra.

#### 3.1.2. Synthesis

A mixture of **1** (1.0 mmol, 0.22 g), thiosemicarbazide, and semicarbazide.HCl (1.1 mmol, 0.1 g, and 0.12 g respectively) was grinded and fused on a hotplate for 5 min until all reactants turned to products. The products were purified by recrystallization from DMF/EtOH to **3** and **5,** respectively.

Ethyl (*E*)-3-((2-carbamothioylhydrazineylidene)methyl)-1*H*-indole-2-carboxylate **3**.

There was 81% yield, 0.23 g, and m.p. 229–230 °C. ^1^H NMR (400 MHz, DMSO-*d*_6_): *δ* 1.41 (t, 3 H, *J* = 6.8 Hz, CH_3_), 4.42 (q, 2 H, *J* = 6.8 Hz, OCH_2_), 7.21 (dd, 1H, *J* = 7.2, 7.6 Hz), 7.35 (dd, 1 H, *J* = 7.2, 7.6 Hz), 7.51 (d, 1 H, *J* = 8.0 Hz), 8.17 (brs, 2 H), 8.40 (d, 1 H, *J* = 8.4 Hz), 9.01 (s, 1H, CH=N-), 11.58 (s, 1H, NH), and 12.15 (s, 1 H, NH indole); ^13^C NMR (100 MHz, DMSO-*d*_6_): *δ* 14.69 (CH_3_), 61.60 (OCH_2_), 113.06, 115.69, 122.32, 124.57, 124.69, 126.12, 127.90, 137.04, 141.10 (9 C), 161.41 (C=O), and 177.97 (C=S); and elemental analysis calculated for [C_13_H_14_N_4_O_2_S]: C, 53.78; H, 4.86; N, 19.30; S, 11.04; found C, 53.89; H, 4.93; N, 19.23; and S, 11.09

Diethyl 3,3’-((1*E*,1’*E*)-hydrazine-1,2-diylidene*bis*(methaneylylidene))*bis*(1*H*-indole-2-carboxylate) **5.**

There was 78% yield, 0.34 g, and m.p. 302–303 °C. ^1^H NMR (400 MHz, DMSO-*d*_6_): *δ* 1.45 (t, 3 H, *J* = 6.4 Hz, CH_3_), 4.48 (q, 2H, *J* = 6.4 Hz, OCH_2_), 7.29 (dd, 1 H, *J* = 6.8, 7.2 Hz), 7.41 (dd, 1 H, *J* = 7.2, 6.8 Hz), 7.58 (d, 1 H, *J* = 7.6 Hz), 8.57 (d, 1 H, *J* = 8.0 Hz), 9.59 (s, 1 H, CH=N), and 12.37 (s, 1 H, NH indole); ^13^C NMR (100 MHz, DMSO-*d*_6_): *δ* 14.67 (CH_3_), 61.70 (OCH_2_), 113.39, 116.09, 122.52, 124.53, 125.34, 126.25, 128.92, 137.07 (8 C), 157.27 (C=O), and 161.24 (C=O); elemental analysis computed for [C_24_H_22_N_4_O_4_]: C, 66.97; H, 5.15; N, 13.02; found C, 66.99; H, 5.21; and N, 13.13. 

Ethyl (*E*)-3-((2-carbamoylhydrazineylidene)methyl)-1*H*-indole-2-carboxylate **6.**

A mixture of **1** (1.0 mmol) and semicarbazide.HCl (1.1 mmol) was refluxed in equal volumes of MeOH/AcOH 10 mL for 8 h until all reactants formed products. A precipitate was formed upon cooling, which was filtered, dried, and recrystallized from MeOH to obtain **6**. 

There was 88% yield, 0.25 g, and m.p. 285–286 °C. ^1^H NMR (400 MHz, DMSO-*d*_6_): *δ* 1.36 (t, *J* 7.1 Hz, 3 H), 4.37 (q, *J* = 7.1 Hz, 2 H), 6.46 (s, 2 H), 7.19 (t, *J* = 7.5 Hz, 1 H), 7.47–7.32 (m, 2H), 7.55 (d, *J* = 8.3 Hz, 1 H), 7.66 (s, 1 H), 8.67 (s, 1 H), and 12.31 (s, 1 H, NH indole ); ^13^C NMR (101 MHz, DMSO-*d*_6_): *δ* 14.63, 61.45, 112.10, 113.63, 121.50, 121.95, 124.78, 125.65, 126.23, 134.06, 136.85, 156.88, and 161.24; elemental analysis calculated for [C_13_H_14_N_4_O_3_]: C, 56.93; H, 5.15; N, 20.43; found C, 57.01; H, 5.13; and N, 20.52.

Ethyl (*E*)-3-(((3-(1*H*-indol-2-yl)-5-thioxo-1,5-dihydro-4*H*-1,2,4-triazol-4-yl)imino)methyl)-1*H*-indole-2-carboxylate **8.**

There was 81% yield, 0.36 g, and m.p. 248–249 °C. ^1^H NMR (400 MHz, DMSO-*d*_6_): *δ* 1.41 (t, 3 H, *J* = 6.8 Hz,CH_3_), 4.46 (q, 2 H, *J* = 6.8 Hz,OCH_2_), 7.02 (dd, 1H, *J* = 7.2, 7.6 Hz), 7.20–7.23 (m, 2H), 7.31 (dd, 1H, and *J* = 7.6 Hz), 7.44–7.52 (m, 3 H), 7.65 (d, 1 H, *J* = 8 Hz), 8.48 (d, 1 H, *J* = 8 Hz), 10.41 (s, 1 H), 11.88 (s, 1 H), 12.79 (s, 1 H, NH indole), and 14.20 (brs, 1 H, NH_trz_); ^13^C NMR(100 MHz, DMSO-*d*_6_): *δ* 14.60 (CH_3_), 62.17 (CH_2_), 105.59, 112.41, 113.11, 113.86, 120.45, 121.55, 123.10, 123.53, 123.86, 124.19, 125.09, 126.61, 127.73, 131.19, 137.12, 137.41, 144.01, 160.84, 162.73, and 163.31. Calculated elemental analysis for [C_22_H_18_N_6_O_2_S]: found C, 61.44; H, 4.37; N, 19.43; S, 7.39; C, 61.38; H, 4.21; N, 19.52; O, 7.43; and S, 7.45

Ethyl (*E*)-3-((2-(4-nitrophenyl)hydrazineylidene)methyl)-1*H*-indole-2-carboxylate **10.**

There was 89% yield, 0.32 g, and m.p. 270–271 °C. ^1^H NMR (400 MHz, DMSO-*d*_6_): δ 1.43 (t, *J* = 7.0 Hz, 3 H), 4.44 (q, *J* = 6.9 Hz, 2 H), 7.28–7.11 (m, 2 H), 7.30 (d, *J* = 7.4 Hz, 1 H), 7.40 (t, *J* = 7.6 Hz, 1 H), 7.54 (d, *J* = 8.2 Hz, 1 H), 8.18 (d, *J* = 8.6 Hz, 2 H), 8.45 (d, *J* = 8.1 Hz, 1 H), 8.99 (s, 1 H), 11.39 (s, 1 H), and 12.13 (s, 1 H, NH indole); ^13^C NMR (101 MHz, DMSO-*d*_6_): *δ* 14.76, 61.44, 111.43, 113.29, 117.04, 122.20, 124.15, 124.56, 126.23, 126.81, 137.10, 138.40, 139.67, 151.01, and 161.46; elemental analysis calculated for [C_18_H_16_N_4_O_4_]: C, 61.36; H, 4.58; N, 15.90; found C, 61.47; H, 4.43; and N, 15.82. 

Ethyl (*E*)-3-((2-(2,4-dinitrophenyl)hydrazineylidene)methyl)-1*H*-indole-2-carboxylate **12.**

There was 89% yield, 0.36 g, and m.p. 292–293 °C. ^1^H NMR (400 MHz, DMSO-*d*_6_): *δ* 1.44 (t, 3 H, CH_3_), 4.46 (q, 2 H, OCH_2_), 7.30–7.54 (m, 3 H), 8.00 (brs, 1 H), 8.38 (brs, 2 H), 8.83 (brs, 1 H), 9.36 (s, 1 H, CH=N), 11.73 (s, 1 H, NH), and 12.33 (s, 1 H, NH indole); ^13^C NMR (100 MHz, DMSO-*d*_6_): *δ* 14.72 (CH_3_), 61.67 (OCH_2_), 113.49, 115.73, 122.76, 123.89, 126.33, 130.43, 137.08, 146.62, and 161.20; elemental analysis calculated for [C_18_H_15_N_5_O_6_]: C, 54.41; H, 3.81; N, 17.63; found C, 54.53; H, 3.88; and N, 17.49. 

#### 3.1.3. X-ray Structure Determination 

The general protocol for the collection of crystalline compounds **3** and **5** is provided in the supporting materials [36,37,38].

## 4. Cytotoxicity

The National Research Institute in Egypt donated the breast cancer (MCF-7) cells, which were collected and cultured in RPMI-1640 medium L-glutamine (Lonza Verviers SPRL, Verviers, Belgium, cat#12-604F). The 10% fetal bovine serum (FBS; Sigma-Aldrich, St. Louis, MO, USA) and 1% penicillin-streptomycin (Lonza, Belgium) were given to each of the two cell lines. 

All cells were cultured, following routine tissue culture work, in 5% CO_2_ humidified at 37 °C. Cells were exposed to compounds at concentrations of 0.01, 0.1, 1, 10, and 100 μM on the second day of culturing. After 48 h, cell viability was evaluated using the MTT solution (Promega, Madison, WI, USA) [38]. MTT dye (20 μL) was placed into each well, and the plate was then incubated for three hours. Absorbance was subsequently measured at 570 nm using the ELISA microplate reader (BIO-RAD, model iMark, Tokyo, Japan), and the percentage of cell viability was calculated, compared to control, as (mean absorbance of tested compound)/(mean absorbance in control) × 100. Finally, IC_50_ values were found using the nonlinear dose–response sigmoidal curve in GraphPad Prism 7 [39].

### 4.1. Investigation of Apoptosis 

Annexin V/PI staining and cell cycle analysis 3–10^5^ MCF-7 cells were added to 6-well culture plates, which were then placed in the incubator for the night. Following that, cells were treated for 48 h to compound **5** at its IC_50_ levels. Following that, PBS was rinsed with ice-cold water before cells and media supernatants were gathered. The cells were then treated with “Annexin V-FITC solution (1:100) and propidium iodide (PI)” at a concentration of 10 g/mL for 30 min in the dark after being suspended in 100 L of annexin-binding buffer solution, which was composed of 25 mM CaCl_2_, 1.4 M NaCl, and 0.1 M Hepes/NaOH, pH 7.4. Then, labeled cells were collected using the Cytoflex FACS system. The data were assessed using the cytExpert program [39].

### 4.2. Wound-Healing Assay (Scratch Assay)

The wound-healing test was mentioned in previous research [40,41]. Six-well plates containing starvation media were filled with four 105 MCF-7 cells per well, and the plates were subsequently incubated at 37 °C for the whole night. A sterile 1 mL pipette tip was used to generate a scratch of the cell monolayer once it was established the following day that the cells had adhered to the well and that cell confluence had reached 90%. Starvation media was used to clean the cells before they were removed from the plates. For 48 h, the cells were cultivated in a CO_2_ incubator with the IC_50_ of compounds **5**, **8**, and **12** in the full medium. After 48 h, the medium was immediately changed to PBS, the wound gap was examined, and cells—both control and treated—were captured on camera with a digital camera attached to an Olympus microscope. The region where the wound closes was measured [42,43].

### 4.3. Kinase Inhibitory Assays

EGFR (catalog #40321), CDK2 (catalog #79599), AKT (catalog #78038), PI3K-α (catalog #40639), β (catalog #79802), and δ (catalog #40628) kinase inhibitions were conducted using an ELISA kit in accordance with the manufacturer instructions from Bioscience, USA. To assess the inhibitory potency of compound **5** against the kinase activity, kinase inhibitory tests were carried out. The following calculation was used to compute the proportion that chemicals inhibited autophosphorylation: 100 − [(A _Control_)/(A _Treated_) − A _Control_]. Using the GraphPad prism7 program, the IC_50_ was calculated using the curves of percentage inhibition of five concentrations of each chemical [44].

### 4.4. In Vivo (SEC-Bearing Model)

The Suez Canal University Research Ethics Committee gave the experimental procedure their seal of approval (approval number REC219/2023, Faculty of Science, Suez Canal University) [45,46]. The full, detailed methodology is supported in the Supplementary Materials. 

### 4.5. Molecular Docking 

Maestro was used to construct, optimize, and energetically favor ligand structures. The X-ray crystallographic structures of EGFR kinase (PDB ID: 1M17), PI3K (PDB = 1E7V), and CDK2 (PDB = 2A4L) [47,48] were subjected to a molecular docking investigation using the AutoDock Vina 1.2.0 software following routine work, which was followed by the Chimera-UCSF 1.17.3 software.

## 5. Conclusions

Ethyl 3-formyl-1*H*-indole-2-carboxylate **1** serves as a precursor in a series of condensation reactions under fusion conditions, aiming to discover novel bioactive lead compounds. Structure assignments were accomplished through NMR and X-ray single-crystal analysis. The majority of the synthesized compounds exhibited noteworthy anti-breast cancer activity. Compounds **5**, **8**, and **12** exhibited superior activity, compared to the standard drug. Compound **5** demonstrated potent cytotoxicity, with IC_50_ values of 2.73 ± 0.14 μM, surpassing staurosporine (IC_50_ = 8.32 ± 0.43 μM) by three-fold. Additionally, it exhibited significant wound-healing activity, with a wound-closure percentage of 48.8%. Notably, in terms of apoptosis induction, compound **5** markedly increased cell death through apoptosis by inhibiting PI3K-α, PI3K-β, PI3K-δ, CDK2, AKT-1, and EGFR kinases, compared to their respective standard drugs. Consequently, the newly identified lead compound is recommended for further development as a kinase-targeted anti-breast cancer agent.

## Figures and Tables

**Table 1:** Crystals data of compounds **3** and **5**.

	3	5
CCDC	2293777	2293778
empirical formula	C_13_H_14_N_4_O_2_S	C_24_H_22_N_4_O_4_
fw	290.34	430.45
temp (K)	120(2)	120(2)
*Λ* (Å)	1.54184	0.71073
cryst syst	*Monoclinic*	*Triclinic*
space group	*P2_1_/c*	*P* *1*
*a* (Å)	9.23490(10)	5.4774(3)
*b* (Å)	19.4168(2)	9.2031(5)
*c* (Å)	7.89280(10)	10.6234(8)
*A* (deg)	90	93.995(5)
*β* (deg)	103.8110(10)	94.008(6)
*γ* (deg)	90	94.201(5)
*V* (Å^3^)	1374.36(3)	531.26(6)
Z	4	1
*ρ*_calc_ (Mg/m^3^)	1.403	1.345
*μ*(Mo K*α*) (mm^−1^)	2.168	0.094
No. reflns.	16497	8485
Unique reflns.	2884	2637
Completeness to θ = 67.684°	99.8%	
Completeness to θ = 25.242°		99.9%
GOOF (*F*^2^)	1.058	1.046
*R*_int_	0.0187	0.0300
*R*_1_ ^a^ (*I* ≥ 2σ)	0.0279	0.0469
*wR*_2_ ^b^ (*I* ≥ 2σ)	0.0780	0.1123

^a^ *R*_1_ = Σ||*F*_o_| − |*F*_c_||/Σ|*F*_o_|. ^b^ *wR*_2_ = {Σ[*w* (*F*_o_^2^ − *F*_c_^2^)^2^]/Σ[*w* (*F*_o_^2^)^2^]}^1/2^.

**Table 2:** Cytotoxicities of the investigated compounds against MCF-7 cells using the MTT assay.

Compounds	IC_50_ ± SD [μM]
**1**	19.7 ± 2.31
**3**	10.2 ± 0.53
**5**	2.73 ± 0.14
**6**	9.42 ± 0.57
**8**	4.38 ± 0.23
**10**	25.4 ± 1.54
**12**	7.03 ± 0.37
**Staurosporine**	8.32 ± 0.43

IC_50_ values were calculated using “Mean ± SD” of three independent values.

**Table 3:** The percentage of wound healing (% closure) for untreated and **5**-treated MCF-7 cells.

Compound	%Closure *, MCF-7
**5**	48.88 ^#^ ± 2.7
**8**	60.74 ^#^ ± 3.43
**12**	51.85 ^#^ ± 2.92
**Untreated control**	94.07 ± 5.5

* Values are expressed as “Mean ± SD”. # Significance level (*p* < 0.05) indicates a significant difference (unpaired Student’s *t*-test) from the untreated control group. Data for length of migration (mm) and area are supported in the Supplementary Materials.

**Table 4:** Flow cytometry results of the three promising cytotoxic agents using Annexin V/PI and DNA-aided flow cytometry.

Compound	Annexin V/PI Staining				DNA Content			
	Total	Early	Late	Necrosis	%G0-G1	%S	%G2/M	%Pre-G1
**Cont.MCF7**	1.27	0.29	0.11	0.87	52.91	41.33	5.76	1.27
**5**	39.26	7.11	22.24	9.91	39.07	56.19	4.74	39.26
**8**	24.38	2.27	13.45	8.66	47.10	46.31	6.57	24.38
**12**	37.05	4.59	17.44	15.02	59.33	38.10	2.55	37.05

**Table 5:** IC_50_ values of kinase activities of the tested compounds.

Compound	IC_50_ [μM] ± SD *					
	PI3K-*α*	PI3K-*β*	PI3K-*δ*	CDK2	AKT-1	EGFR
**5**	1.73 ± 0.1	2.27 ± 0.11	2.68 ± 0.15	0.156 ± 0.01	0.602 ± 0.03	0.058 ± 0.029
**LY294002**	8.52 ± 0.48	0.44 ± 0.02	0.85 ± 0.05	NT	NT	NT
**erlotinib**	NT	NT	NT	0.173 ± 0.01	NT	0.038 ± 0.019
**A-674563**	NT	NT	NT	NT	0.26 ± 0.01	NT

* “Values are expressed as an average of three independent replicates”. “IC_50_ values were calculated using a sigmoidal non-linear regression curve fit of percentage inhibition against five concentrations of each compound”. NT = Not tested.

## Data Availability

Data is contained within the article and supplementary material.

## Supplementary Materials

The following documents are available for download at https://www.mdpi.com/1424-8247/16/12/1724/s1, where you can find the titles of Figures S1–S12: NMR spectrum of the synthesized compounds, Figure S13: Wound-healing assay for the most active compounds. In vivo assay protocol. References [49,50,51,52] are cited in the supplementary materials.
