# Supplementary material for: Discovery of Potent Indolyl-Hydrazones as Kinase Inhibitors for Breast Cancer: Synthesis, X-ray Single-Crystal Analysis, and In Vitro and In Vivo Anti-Cancer Activity Evaluation
Source: Pharmaceuticals (Basel). 2023 Dec 13;16(12):1724. doi: 10.3390/ph16121724 (PMC10748079; doi:10.3390/ph16121724)
Supplement: Supplementary file 1 [file pharmaceuticals-16-01724-s001.zip › pharmaceuticals-2718906-supplementary.pdf]

Supplementary information

Discovery of Potent Indolyl-Hydrazones as Kinase Inhibitors for Breast Cancer: Synthesis, X-ray Single-Crystal Analysis, and In Vitro and In Vivo Anti-Cancer Activity Evaluation

Eid E. Salama [1,*], Mohamed F. Youssef [1], Ahmed Aboelmagd [1], Ahmed T. A. Boraei [1], Mohamed S. Nafie [1,2], Matti Haukka [3], Assem Barakat [4,*] and Ahmed A. M. Sarhan [5]

[1] Department of Chemistry, Faculty of Science, Suez Canal University, Ismailia 41522, Egypt; mohamed_gomaa@science.suez.edu.eg (M.F.Y.); ahmed_mohamed@science.suez.edu.eg (A.A.); ahmed_tawfeek83@yahoo.com or ahmed_boraei@science.suez.edu.eg (A.T.A.B.); mohamed_nafie@science.suez.edu.eg (M.S.N.)
[2] Department of Chemistry, College of Sciences, University of Sharjah, Sharjah P.O. Box 27272, United Arab Emirates
[3] Department of Chemistry, University of Jyväskylä, P.O. Box 35, FI-40014 Jyväskylä, Finland; matti.o.haukka@jyu.fi
[4] Chemistry Department, College of Science, King Saud University, P.O. Box 2455, Riyadh 11451, Saudi Arabia
[5] Chemistry Department, Faculty of Science, Arish University, Al-Arish 45511, Egypt; ahmed_sarhan252@yahoo.com or asarhan@aru.edu.eg
* Correspondence: eid_mohamed@science.suez.edu.eg (E.E.S.); ambarakat@ksu.edu.sa (A.B.); Tel.: +966-11467-5901 (A.B.); Fax: +966-11467-5992 (A.B.)

NMR spectroscopy

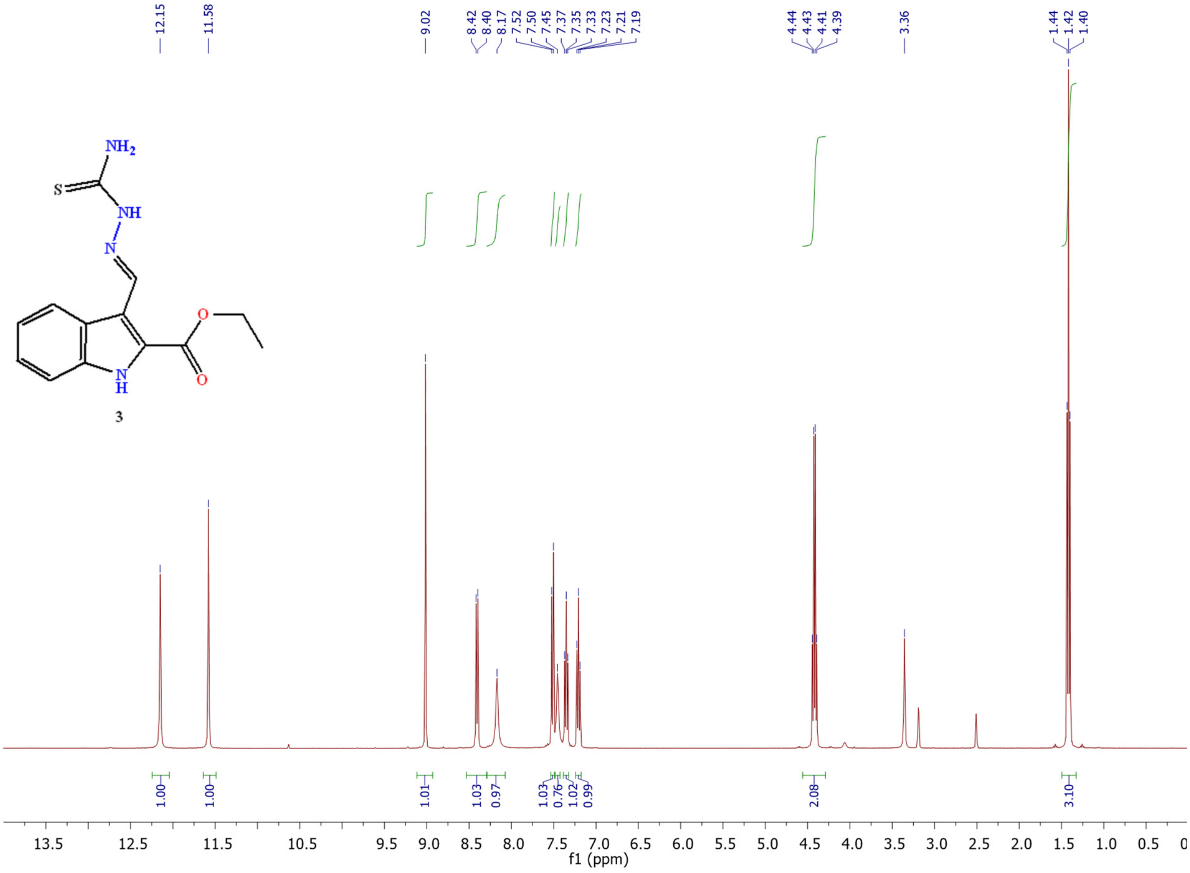

Figure S1: ^{1}H-NMR of compound **3.**

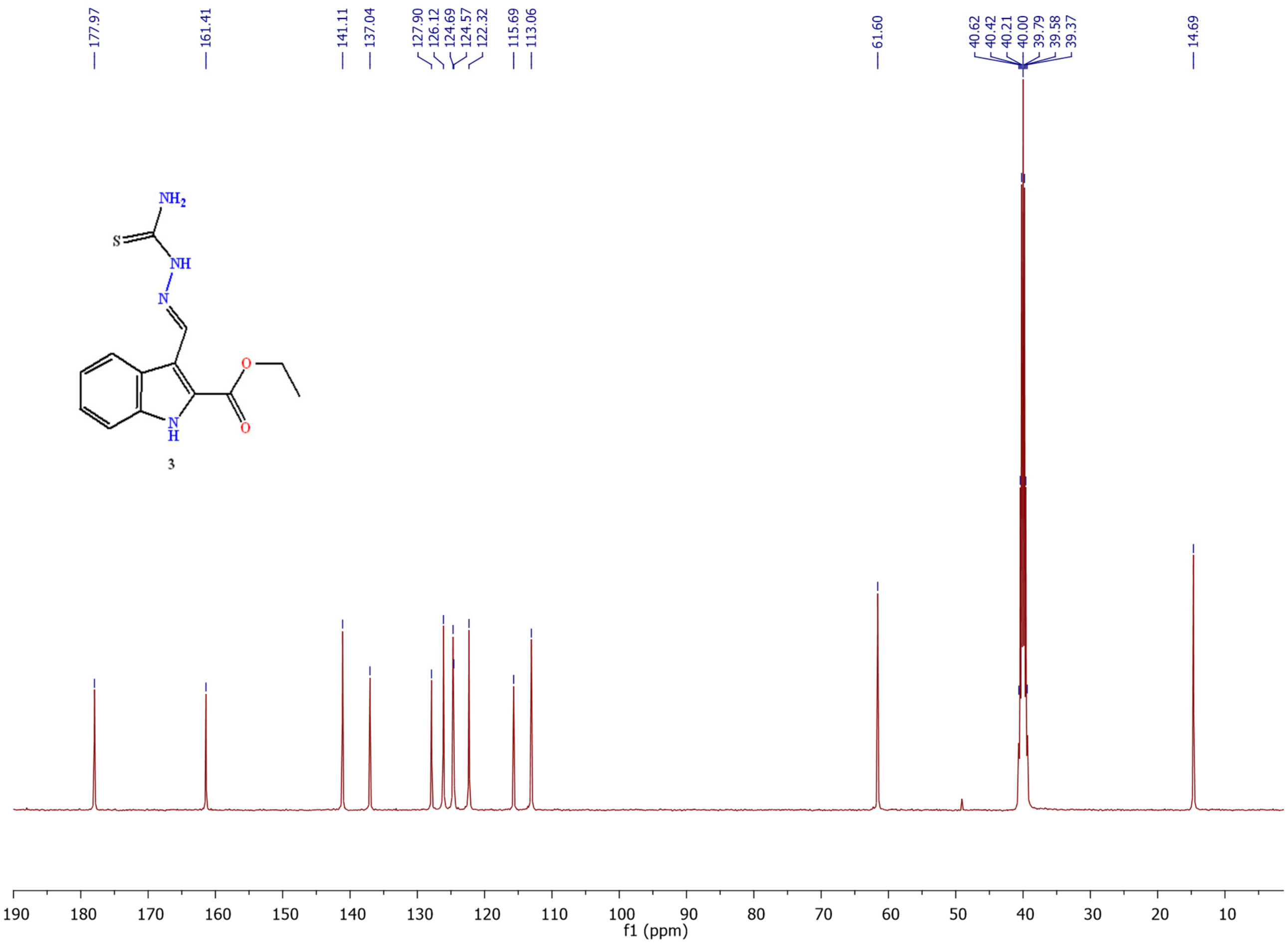

Figure S2: ^{13}C-NMR of compound **3.**

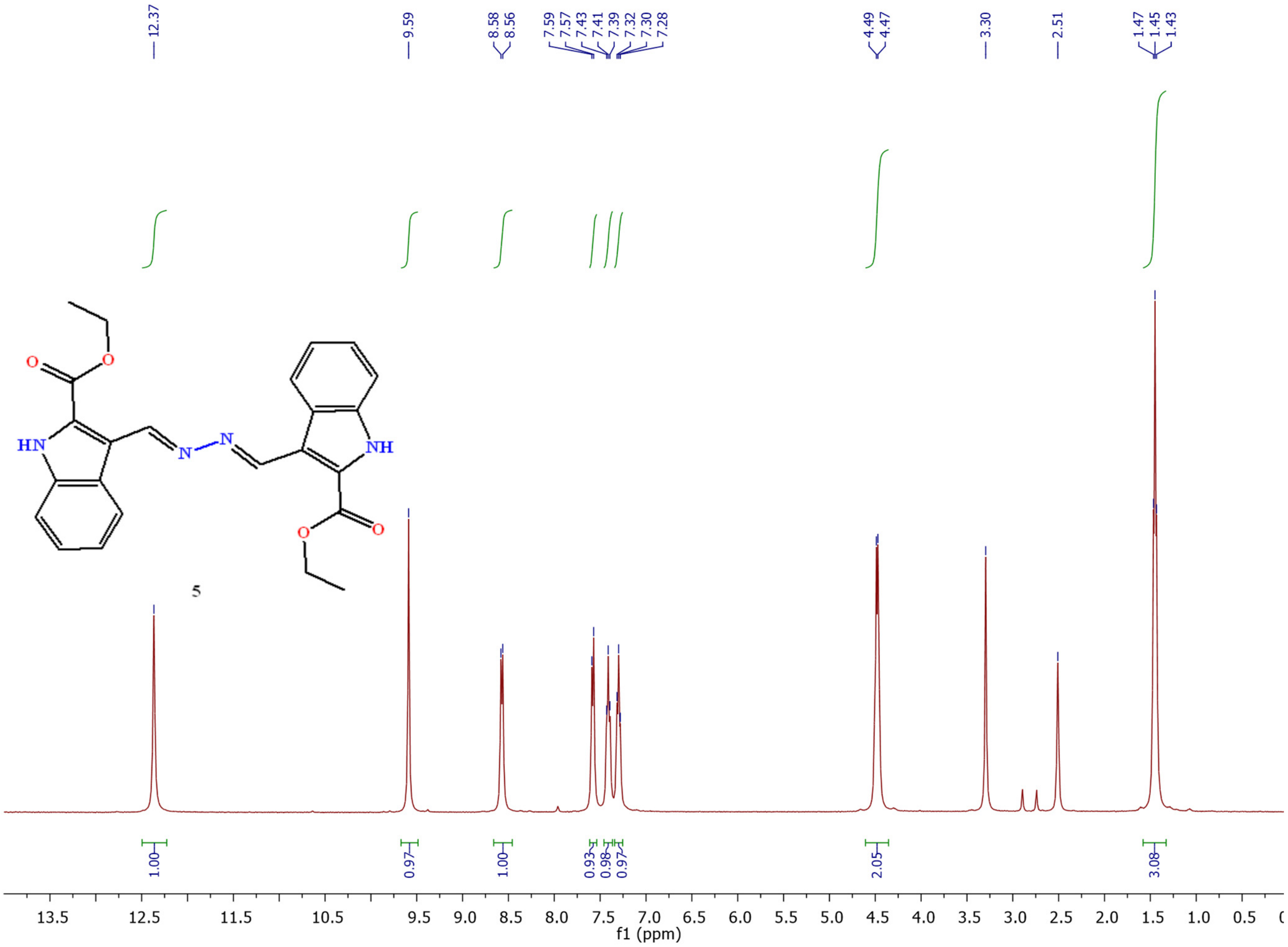

Figure S3: ^{1}H-NMR of compound **5.**

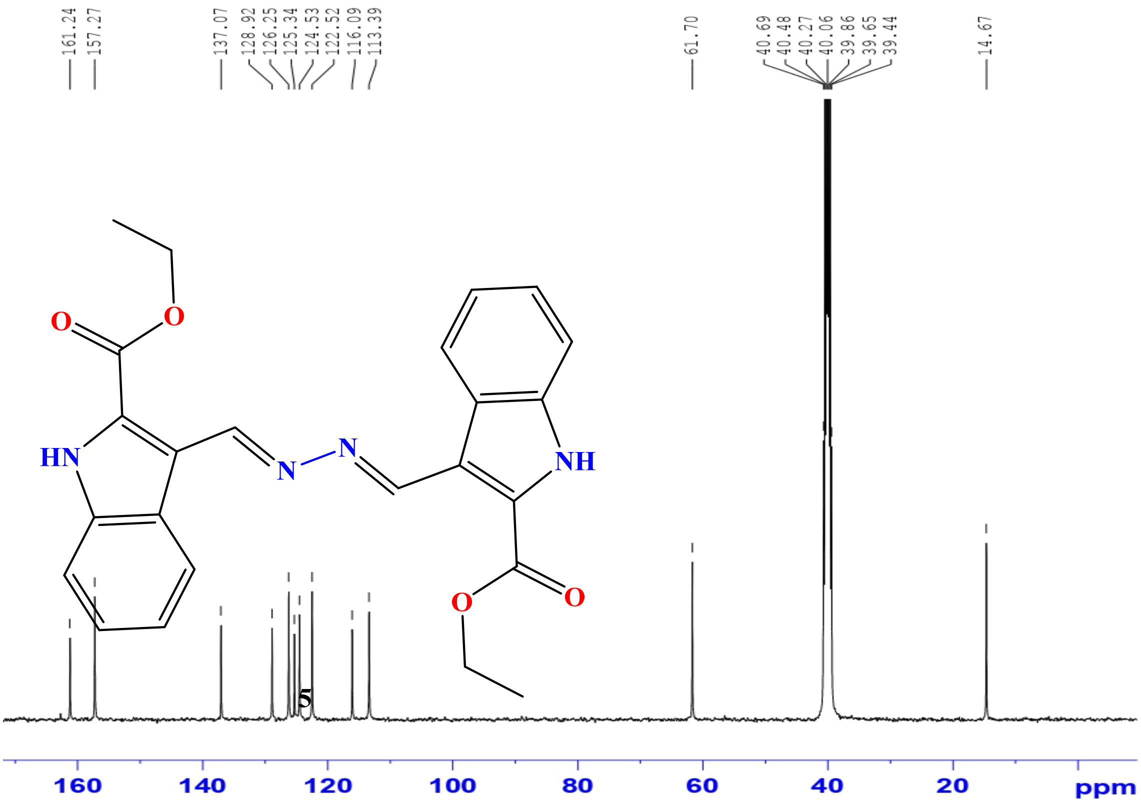

Figure S4: ^{13}C-NMR of compound **5.**

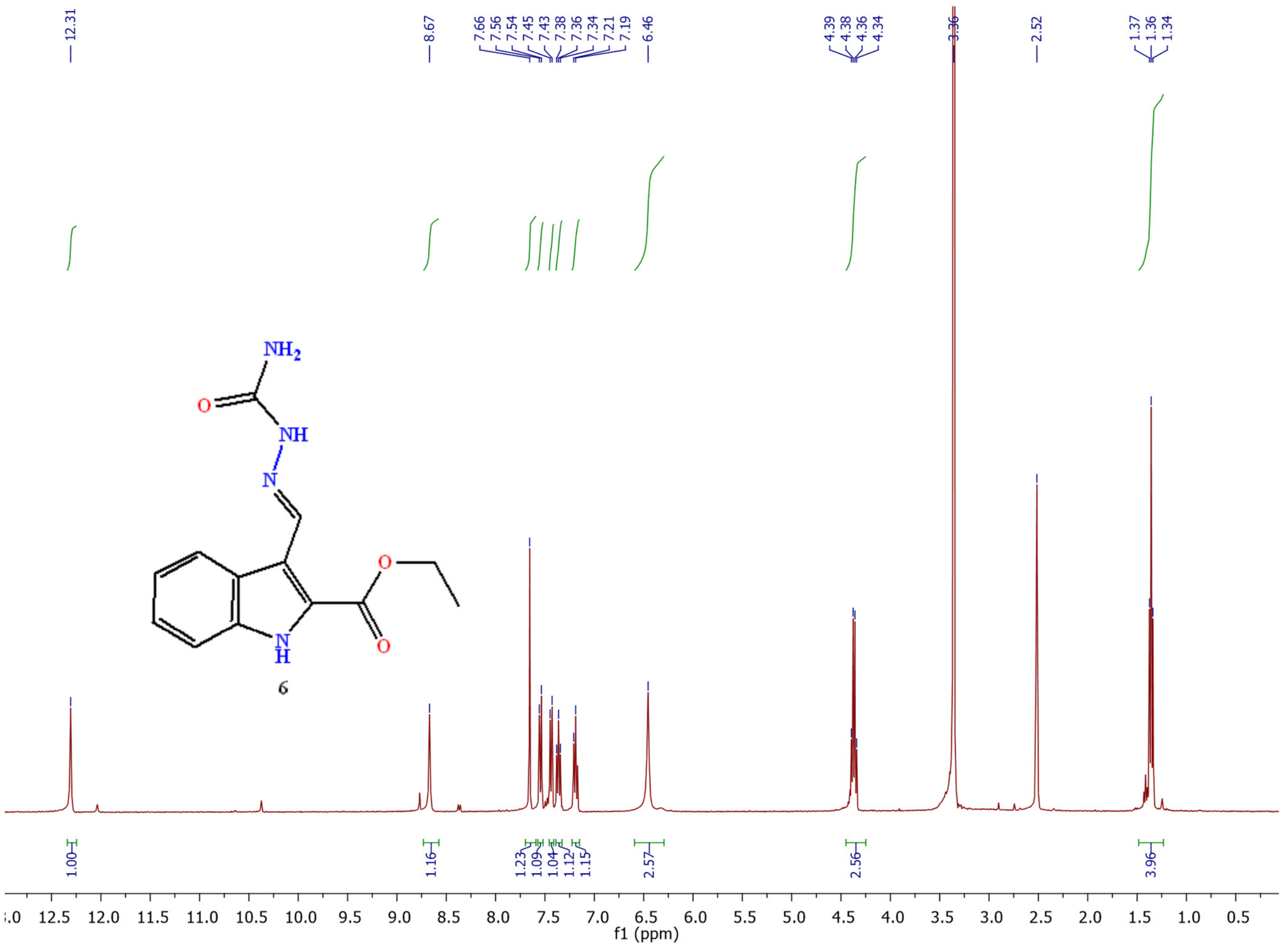

Figure S5: ^{1}H-NMR of compound **6.**

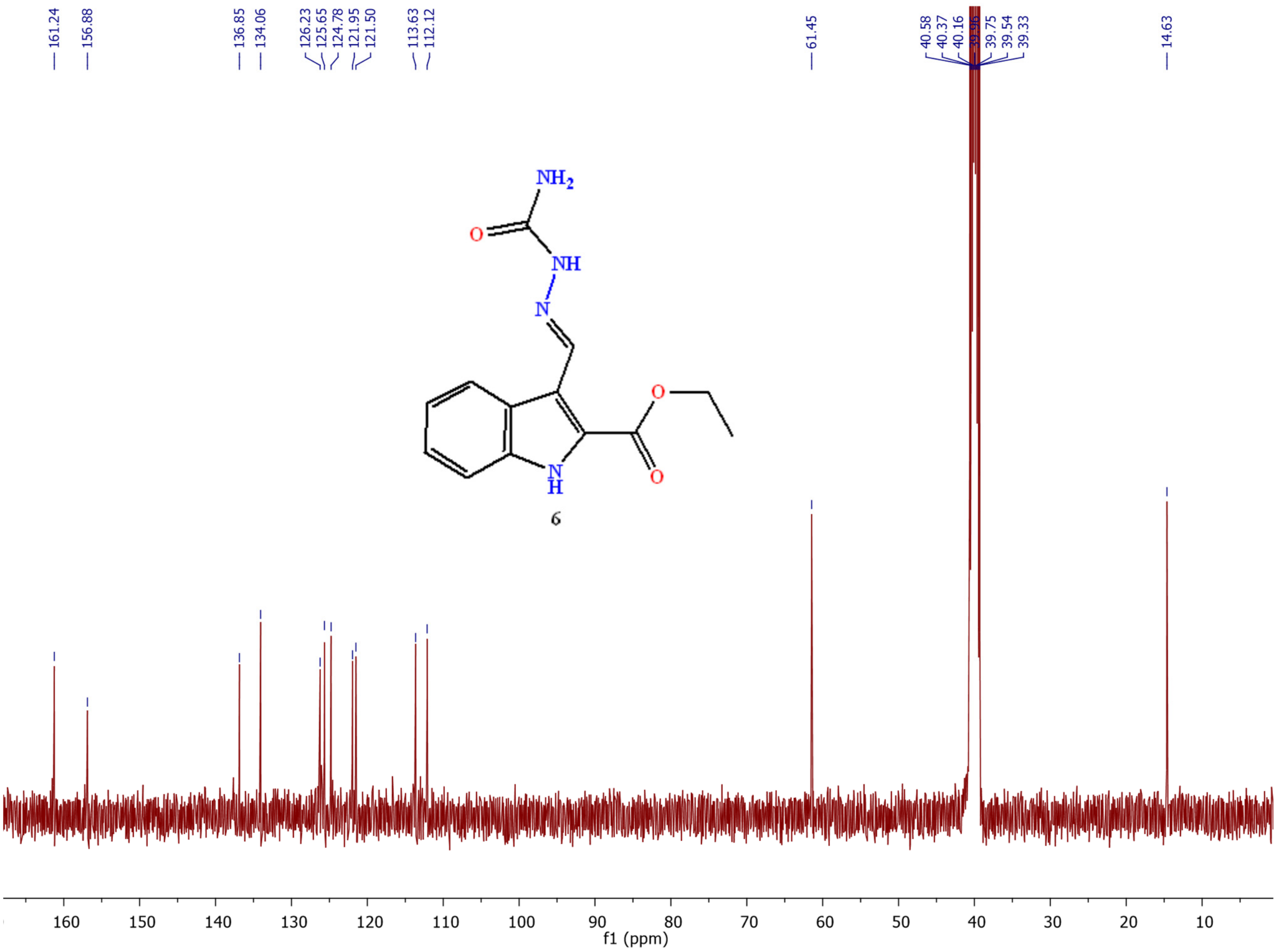

Figure S6: ^{13}C-NMR of compound **6.**

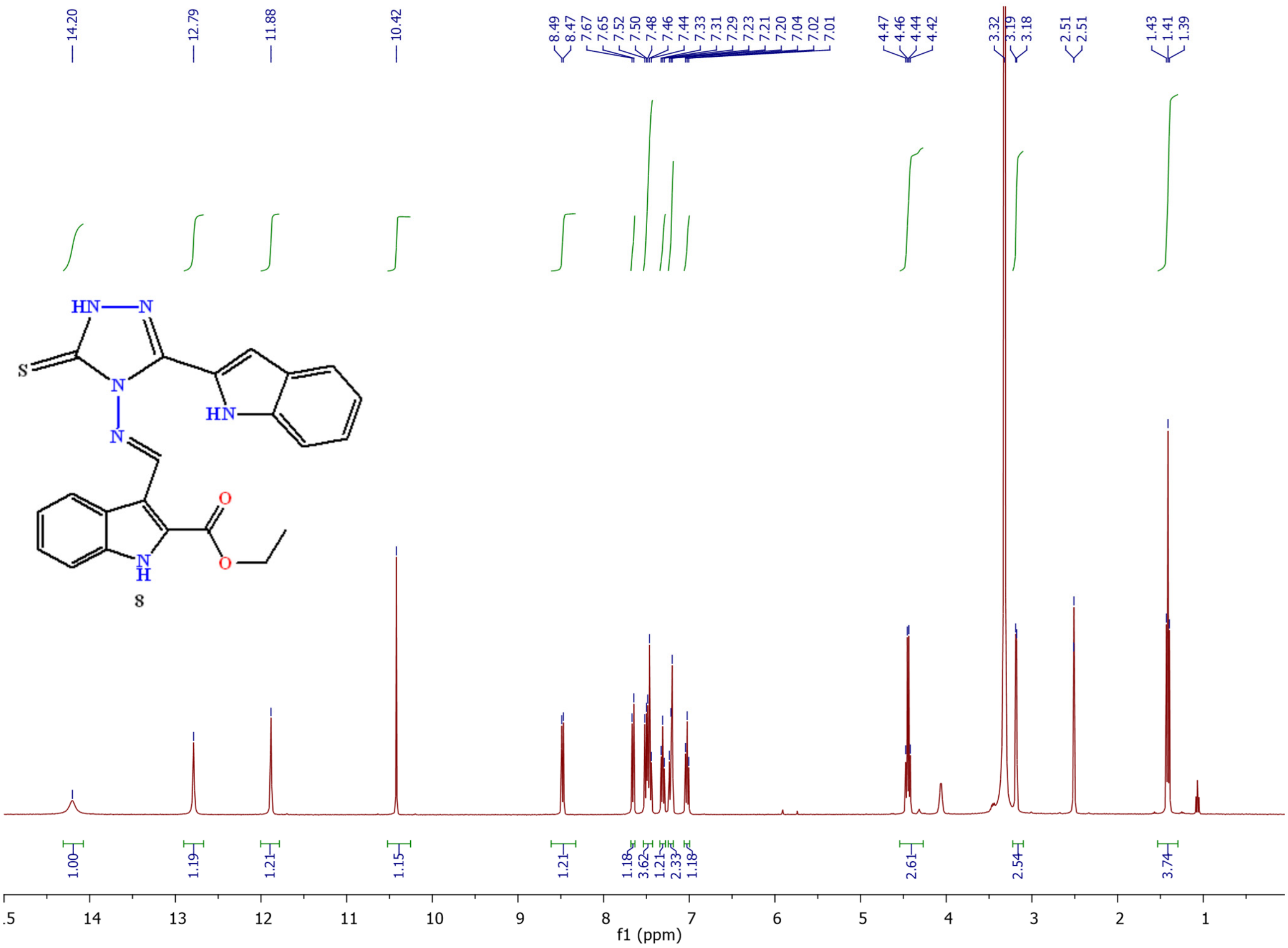

Figure S7: ^{1}H-NMR of compound **8.**

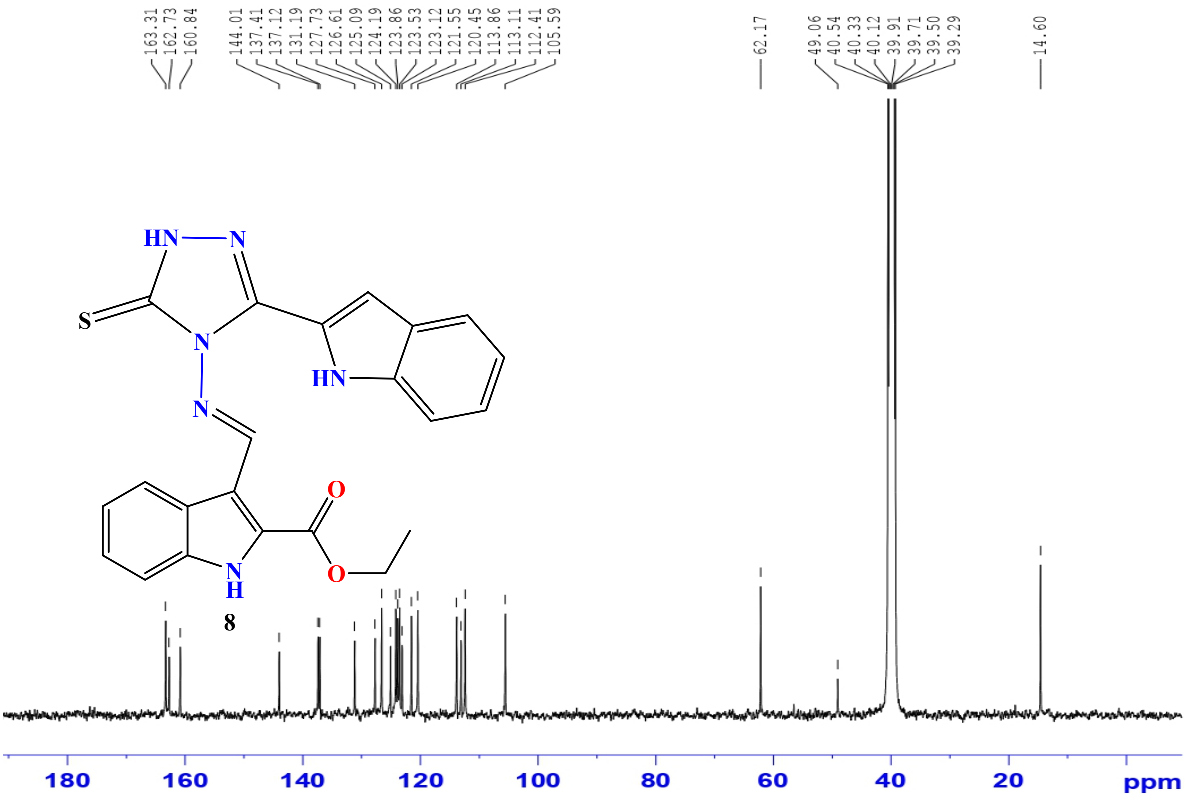

Figure S8: ^{13}C-NMR of compound **8.**

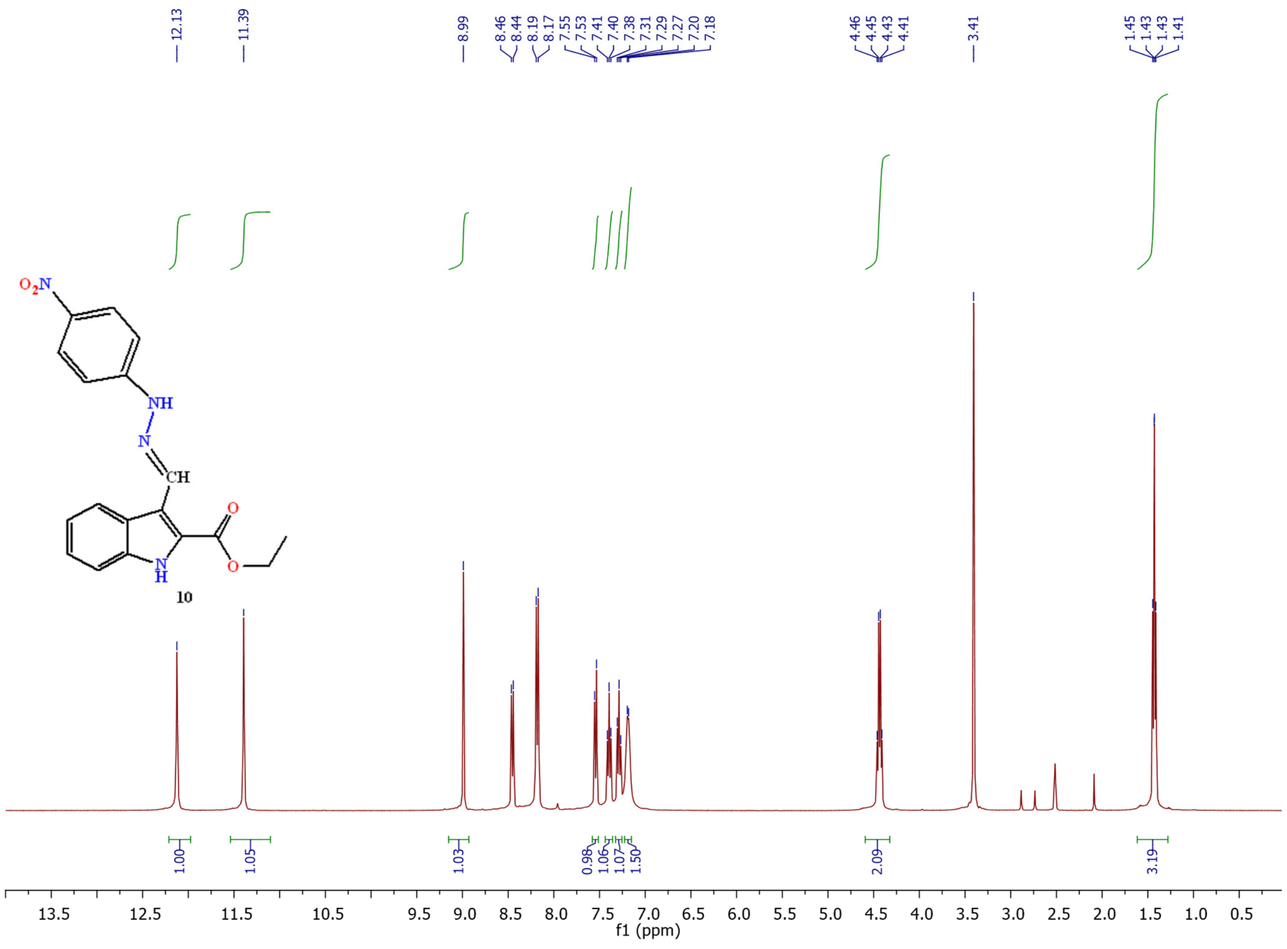

Figure S9: ^{1}H-NMR of compound **10.**

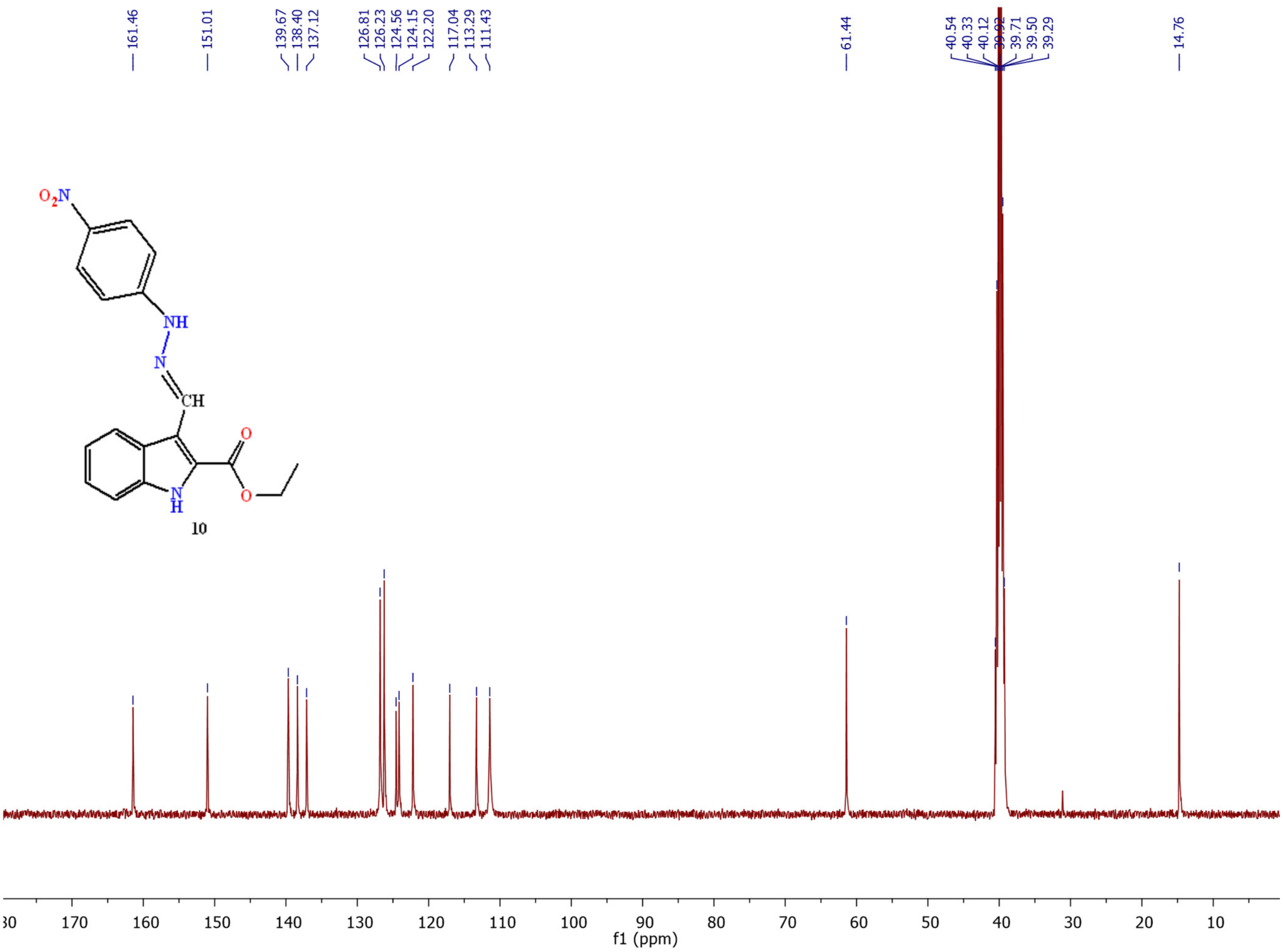

Figure S10: ^{13}C-NMR of compound **10.**

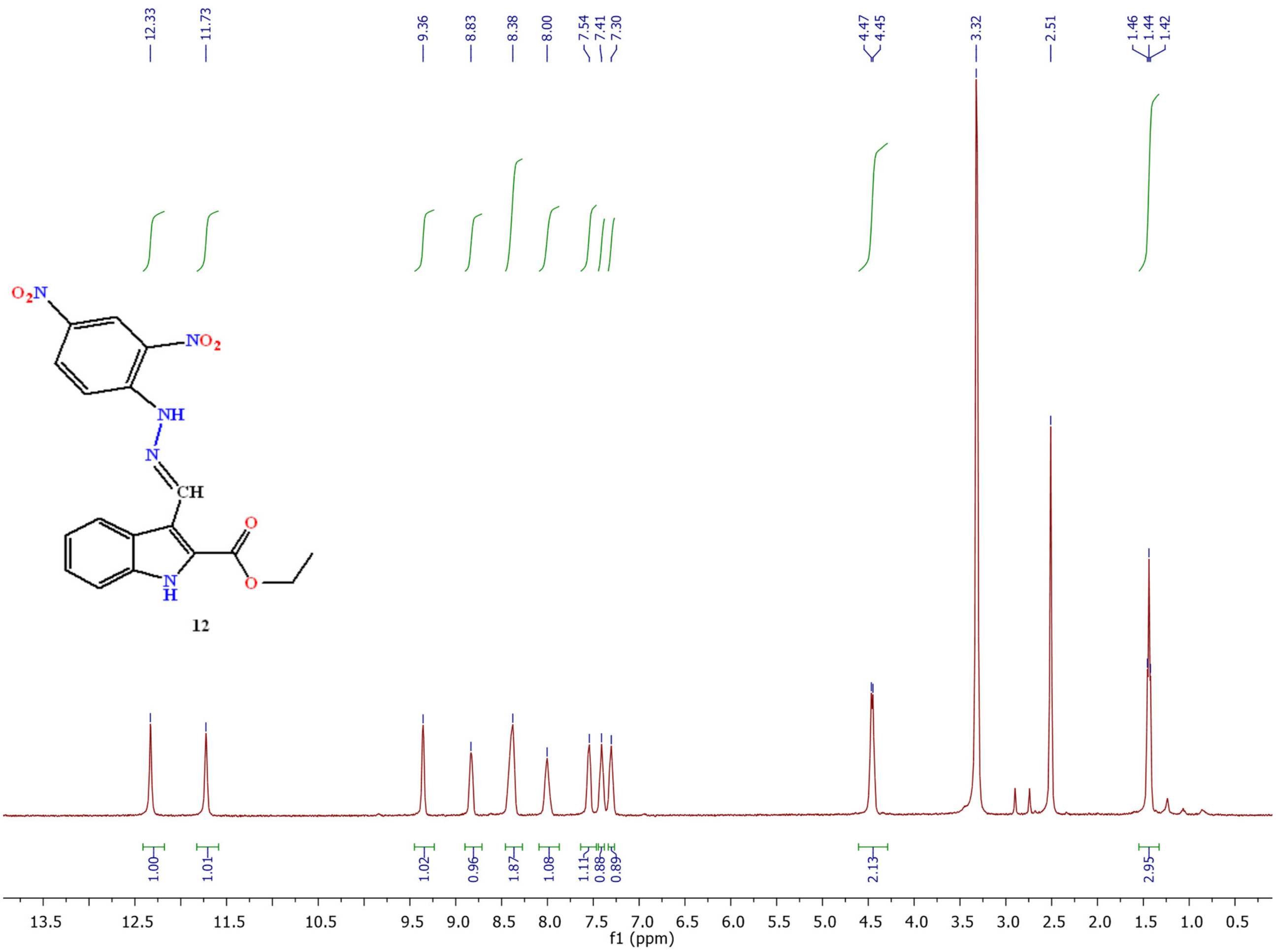

Figure S11: ^{1}H-NMR of compound **12.**

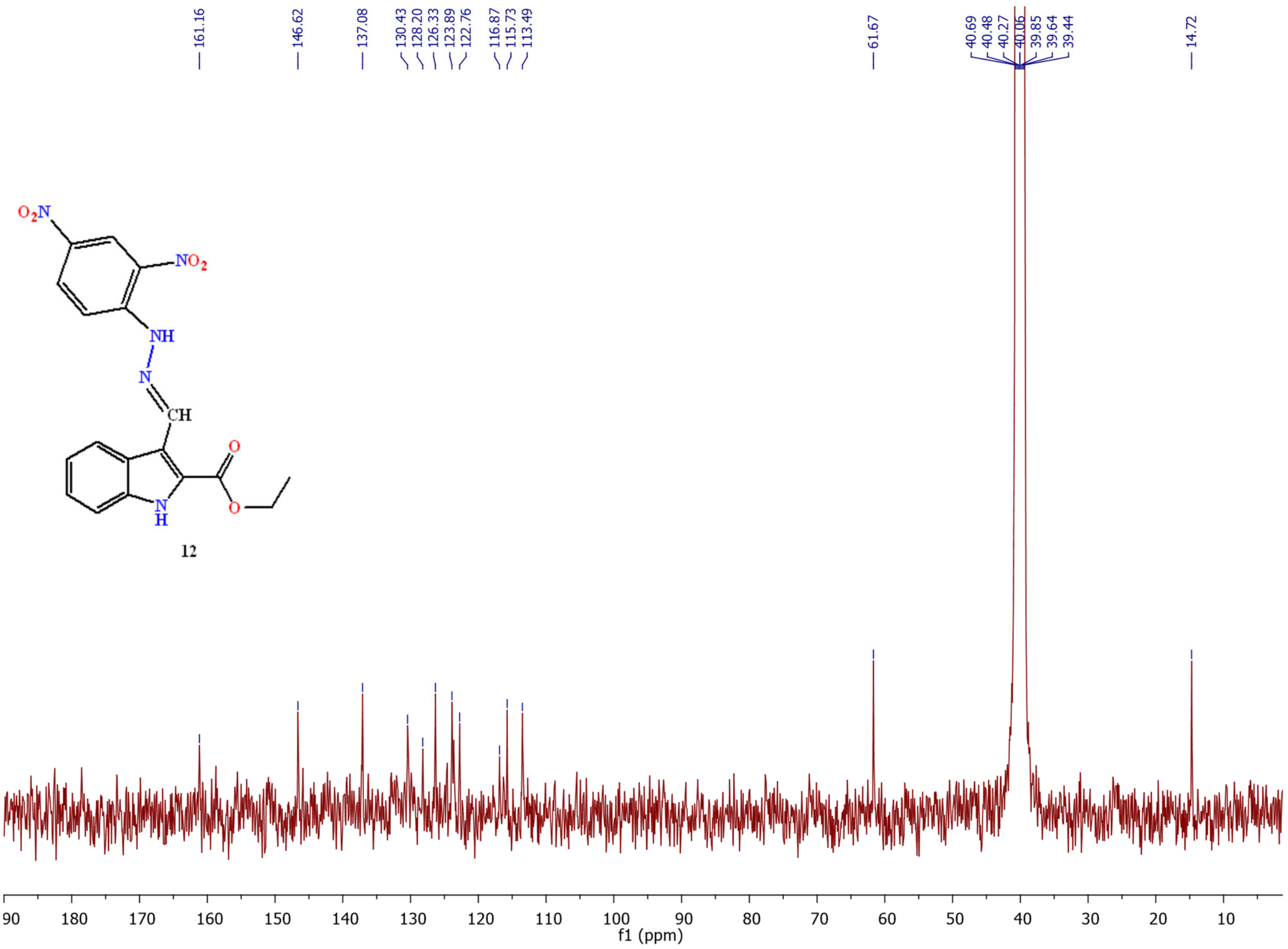

Figure S12: ^{13}C-NMR of compound **12.**

Wound healing assay

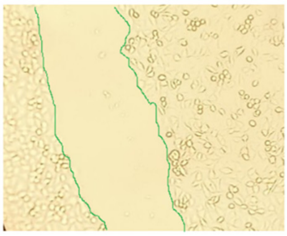

An-59/MCF7

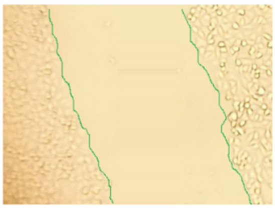

scratch control MCF7

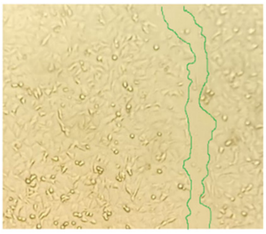

control MCF7

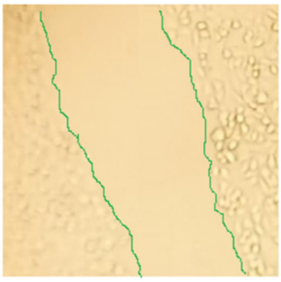

An-60/MCF7

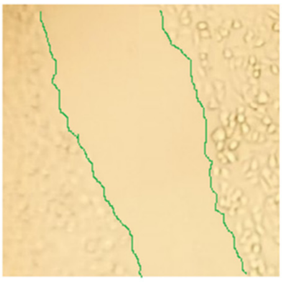

An-60/MCF7

ser	Sample				wound healing	SD
	code	MW	cells	conc uM	% Closure	±
2	An-59 = 8	430.12			60.741	3.43
3	An-60 = 12	397.10			51.852	2.92
4	control	---			94.074	5.31

**	% Closure	Total area	Migrated cells area	T 72h	Length mm	L.of migration ΔL	L1	L2	L3
An-59/MCF7	60.741	0.198	0.1202667		0.22	0.27	0.28	0.26	0.28
An-60/MCF7	51.852	0.198	0.1026667		0.22	0.23	0.22	0.25	0.23
control.MCF7	94.074	0.198	0.1862667		0.22	0.42	0.42	0.41	0.44

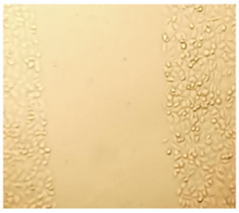

An-40/MCF7

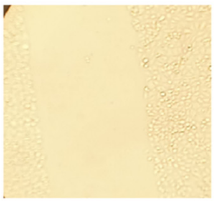

An-40/MCF7

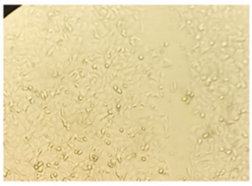

Control MCF7

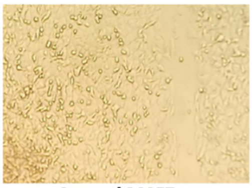

Control MCF7

ser	Sample				wound healing Results MCF7	
	code	MW	cells	IC50	% Closure	
2	An-40 = 5	216.09	MCF7	2.73	48.88±2.7	
3	control	---	---	---	97.77±5.5	

	**	% Closure	Total area	Migrated cells area	T 72h	Length mm	L.of migration ΔL	L1	L2	L3
2	An-40	48.889	0.369	0.1804		0.41	0.22	0.42	0.39	0.43
3	control	97.778	0.369	0.3608		0.41	0.44	0.42	0.39	0.43

Figure S13: Wound healing for the most active compounds.

In Vivo Assay

Animals and tumor cell line

Adult female Swiss albino mice purchased from Theodor Bilharzia Research Institute, Giza, Egypt, with an average body weight of (18-23) g was used. Mice were housed under constant conditions of 12 h light/dark cycle in a temperature under conditions of controlled humidity (22 ± 2 °C), with free access to standard laboratory mice food and water .All procedures related to care and maintenance of the animals were performed according to the international guiding principles for animal research and approved by Faculty of Science, Suez Canal University bioethics and animal ethics committee (Approval number REC219/2023).

Solid Ehrlich carcinoma (SEC) were got from the National Cancer Institute (Cairo University, Egypt). The tumor cell line was proliferated in mice through serial intraperitoneal (I.P.) transplantation of a volume of 0.2 mL physiological saline contains 1 × 10^6 viable cells for 24 h. SEC cells were collected 7 days after I.P. implantation. The harvested cells were diluted with saline to obtain a concentration of 5 × 10^6 viable SEC cells/mL. A volume of 0.2 mL saline contains 1 × 10^6 SEC cells that were I.P. implanted into each normal mouse. SEC cells (1 × 10^6 tumor cells/mouse) were implanted subcutaneously into the right thigh of the hind limb.

The experimental animals were randomly divided into four groups. Group 1 served as the normal saline control (5 mL/kg B.Wt., I.P.). Group 2 served as the SEC control (1 × 10^6 cells/mouse). Group 3 served as the compound-treated group (5 mg/kg B.Wt., I.P.). Group 4 received the standard anticancer drug of Doxorubicin (DOX) (5 mg/kg BW, I.P.) and is considered as a reference control. Body weight and survival were recorded daily until the 24th day in both treated and control groups. At the end of experiment, the blood of each group was collected under light anesthesia for estimation of hematological and biochemical assays. The anesthetized animals were then sacrificed for evaluation of the antitumor activity and histopathological examination.

Antitumor potentiality

It includes tumor volume, weight, and tumor inhibition ration (TIR%). Time interval measurements of tumor volume using digital Vernier caliper (Tricle Brand, Shanghai,

China). Measure tumor length and width using clipper and then calculate tumor volume using formulations V = (L x W x W)/2, where V is tumor volume, W is tumor width, L is tumor length. While TIR% was calculated according to the following equation $\frac{Tumor\ volume\ (Control) - Tumor\ volume\ (treated)}{Tumor\ volume\ (control)} x\ 100$.

Blood assays

At the end of the experiment, animals from different groups were sacrificed, and blood samples were collected for determination of liver enzymes ALT, AST levels. Activities of aspartate aminotransferase (AST) and alanine aminotransferase (ALT) were evaluated using commercial kits (ELITech clinical systems, France). Serum albumin level was determined by kit purchased from STANBIO Company (USA).

Histopathological study

Specimens of liver-sacrificed mice were fixed in 10% saline formalin. The fixed liver specimens were dehydratedin ascending series of ethyl alcohol and embedded in paraffin. Sections at 5 mm thicknesses were stained with hematoxylin and eosin and examined under light microscopy.

References

50. Dhanalakshmi, B.; Anil Kumar, B.M.; Srinivasa Murthy, V.; Srinivasa, S.M.; Vivek, H.K.; Sennappan, M.; Rangappa, S. Design, Synthesis and Docking Studies of Novel 4-Aminophenol-1,2,4-Oxadiazole Hybrids as Apoptosis Inducers against Triple Negative Breast Cancer Cells Targeting MAP Kinase. *J. Biomol. Struct. Dyn.* **2023**, *0*, 1–17, doi:10.1080/07391102.2023.2239912
51. Dicato, M.; Plawny, L.; Diederich, M. Anemia in cancer. *Ann. Oncol.* **2010**, *21*, vii167–vii172. https://doi.org/10.1093/annonc/mdq284.
52. HEl Zahabi, S.A.; Nafie, M.S.; Osman, D.; Elghazawy, N.H.; Soliman, D.H.; EL-Helby, A.A.H.; Arafa, R.K. Design, synthesis and evaluation of new quinazolin-4-one derivatives as apoptotic enhancers and autophagy inhibitors with potent antitumor activity. *Eur. J. Med. Chem.* **2021**, *222*, 113609. https://doi.org/10.1016/j.ejmech.2021.113609.
53. Boraei, A.T.A.; Eltamany, E.H.; Ali, I.A.I.; Gebriel, S.M.; Nafie, M.S. Synthesis of new substituted pyridine derivatives as potent anti-liver cancer agents through apoptosis induction: In vitro, in vivo, and in silico integrated approaches. *Bioorganic Chem.* **2021**, *111*, 104877. https://doi.org/10.1016/j.bioorg.2021.104877.
